# Wheat Disease Resistance Genes and Their Diversification Through Integrated Domain Fusions

**DOI:** 10.3389/fgene.2020.00898

**Published:** 2020-08-05

**Authors:** Ethan J. Andersen, Madhav P. Nepal, Jordan M. Purintun, Dillon Nelson, Glykeria Mermigka, Panagiotis F. Sarris

**Affiliations:** ^1^Department of Biology, Francis Marion University, Florence, SC, United States; ^2^Department of Biology and Microbiology, South Dakota State University, Brookings, SD, United States; ^3^Department of Math, Science and Technology, Oglala Lakota College, Kyle, SD, United States; ^4^Department of Biology, University of Crete, Crete, Greece; ^5^Institute of Molecular Biology and Biotechnology, FORTH, Crete, Greece; ^6^School of Biosciences, College of Life and Environmental Sciences, University of Exeter, Exeter, United Kingdom

**Keywords:** wheat R-genes, integrated domain, pathogen resistance, alternative splicing, disease resistance

## Abstract

Plants are in a constant evolutionary arms race with their pathogens. At the molecular level, the plant nucleotide-binding leucine-rich repeat receptors (NLRs) family has coevolved with rapidly evolving pathogen effectors. While many NLRs utilize variable leucine-rich repeats (LRRs) to detect effectors, some have gained integrated domains (IDs) that may be involved in receptor activation or downstream signaling. The major objectives of this project were to identify NLR genes in wheat (*Triticum aestivum* L.) and assess IDs associated with immune signaling (e.g., kinase and transcription factor domains). We identified 2,151 NLR-like genes in wheat, of which 1,298 formed 547 gene clusters. Among the non-toll/interleukin-1 receptor NLR (non-TNL)-like genes, 1,552 encode LRRs, 802 are coiled-coil (CC) domain-encoding (CC-NBS-LRR or CNL) genes, and three encode resistance to powdery mildew 8 (RPW8) domains (RPW8-NBS-LRR or RNL). The expansion of the NLR gene family in wheat is attributable to its origin by recent polyploidy events. Gene clusters were likely formed by tandem duplications, and wheat NLR phylogenetic relationships were similar to those in barley and *Aegilops*. We also identified wheat NLR-ID fusion proteins as candidates for NLR functional diversification, often as kinase and transcription factor domains. Comparative analyses of the IDs revealed evolutionary conservation of more than 80% amino acid sequence similarity. Homology assessment indicates that these domains originated as functional non-NLR-encoding genes that were incorporated into NLR-encoding genes through duplication events. We also found that many of the NLR-ID genes encode alternative transcripts that include or exclude IDs, a phenomenon that seems to be conserved among species. To verify this, we have analyzed the alternative transcripts that include or exclude an ID of an NLR-ID from another monocotyledon species, rice (*Oryza sativa*). This indicates that plants employ alternative splicing to regulate IDs, possibly using them as baits, decoys, and functional signaling components. Genomic and expression data support the hypothesis that wheat uses alternative splicing to include and exclude IDs from NLR proteins.

## Introduction

Plant innate immune systems utilize specialized receptor proteins to detect pathogens (Jones and Dangl, 2006; Duxbury et al., 2016). Nucleotide-binding leucine-rich repeat receptor (NLR) proteins detect pathogen effectors that would otherwise inhibit host resistance responses (Jones et al., 2016). NLRs can be recognized by the domain nucleotide-binding site found in apoptotic protease activating factor 1, resistance genes, and *Caenorhabditis elegans* death-4 protein (NB-ARC) (Mermigka et al., 2020). The NB-ARC is associated with ATP/ADP binding, since the molecule uses it upon activation. In order to detect hundreds of pathogens and pests, immune receptors must be able to respond to many elicitors. To accomplish this, NLRs have radiated to form a diverse family of resistance genes in plants (Shao et al., 2016). Much of this diversity originated via gene duplication and variation in NLR leucine-rich repeats (LRRs), which allows NLRs to bind to new effectors (Deyoung and Innes, 2006). Diversification has led to the formation of networks of sensor and helper NLRs, with some NLRs dimerizing to initiate signaling (Hayashi et al., 2010; Kofoed and Vance, 2011; Bonardi et al., 2012; Bonardi and Dangl, 2012). NLRs have also diversified by gaining extra domains that may facilitate pathogen recognition or resistance signaling. These domains, called integrated domains (IDs), resulted from fusions of NLRs and other functional domains also involved in resistance, as outlined by the integrated decoy/sensor model (Cesari et al., 2014; Wu et al., 2015; Duxbury et al., 2016). Studies of ID diversity across many plant genomes have revealed a diversity of domains associated with potential roles in resistance (Sarris et al., 2016).

Wheat (*Triticum aestivum* L.) provides approximately 20% of the human population’s caloric intake (FAOstat, 2013) and is afflicted by over 100 different diseases caused by various pathogen and pest species (Murray et al., 2015). Diseases that substantially reduce yield, such as biotrophic rusts and necrotrophic leaf spotting diseases, impact global markets and food supplies. Historically devastating pathogens continue to produce new strains, which overcome past sources of resistance, such as in the case of Ug99 stem rust (Saari and Prescott, 1985; Pretorius et al., 2000; Huerta-Espino et al., 2011; Singh et al., 2011). Disease resistant wheat cultivars have improved agricultural productivity and our understanding of phytopathology (Mcfadden, 1930; Borlaug, 1983). Advanced genetic and genomic technologies have been used to identify pathogen resistance genes (R-genes), such as the recently discovered Ug99 R-genes *Sr33* and *Sr35* (Periyannan et al., 2013; Saintenac et al., 2013). The hexaploid bread wheat genome (AABBDD), a draft of which has recently become available (International Wheat Genome Sequencing Consortium, 2014), formed through the hybridization of three separate species: *Triticum urartu* (A), an unknown relative of *Aegilops speltoides* (B), and *Aegilops tauschii* (D) (Jia et al., 2013; Ling et al., 2013; Marcussen et al., 2014). The polyploid origins of wheat may have resulted in novel mechanisms to regulate its multiple progenitor resistance signaling pathways. The large and redundant nature of wheat’s hexaploid genome makes it a good candidate for studying R-gene evolution with respect to recent polyploidization events.

The objectives of this research were to conduct genome-wide identification of wheat R-genes encoding NB-ARC domains, identify wheat NLR-ID fusion proteins, and assess their homology in wheat relatives and other monocot species. Results showed that tandem duplications can explain many of the events that led to the diversification of these genes. We also propose a mechanism to describe how plants carry out NLR-ID regulation, which became apparent while manually assessing the variation among NLR-ID transcripts. This mechanism was investigated using transient expression of a NLR gene from rice (*Oryza sativa* Japonica) in *Nicotiana benthamiana* leaves. Identifying the evolutionary patterns of the NLR-ID fusions improves our understanding of how NLRs diversify to oppose various pathogenic molecular weapons.

## Materials and Methods

### NB-ARC Identification

*Triticum aestivum* chromosome, gene, and protein sequences were downloaded using the Biomart application within the Ensembl Genomes (Kersey et al., 2015) and Phytozome (Goodstein et al., 2011) databases. InterProScan annotations (Jones et al., 2014) were compiled, and proteins containing NB-ARC domain (PF00931) were investigated. The locations of genes encoding NB-ARCs were analyzed for clusters as described in Jupe et al. (2012), who used the criteria that clustered genes must be within 200,000 bases of each other and must be separated by fewer than eight additional genes between them (Jupe et al., 2012). NB-ARC domain motifs were also assessed using MEME software (Bailey et al., 2009), which was used to identify those with P-loop, Kinase-2, and GLPL motifs. Clustered genes with the aforementioned motifs were aligned and manually curated using the ClustalW2 program integrated within the program Geneious (Larkin et al., 2007; Kearse et al., 2012). The program MEGA 7 was used to construct a neighbor-joining tree with 100 bootstraps (Kumar et al., 2016) to assess whether the clustered genes nested together formed by potential tandem duplication. Wheat and *Aegilops tauschii* R-gene locations from Ensembl Genomes were used to construct a genomic map using the program Circa^1^. Clustered wheat R-genes were also compared using the program Circoletto (Darzentas, 2010) to visualize similarities between genes.

### Integrated Domain Identification

Pfam annotations not inherently part of the NLR structure (CC/TIR, NB-ARC, and LRR) were assembled. Amino acid sequences and corresponding annotations were uploaded to the program Geneious (Kearse et al., 2012) for sequence alignment, homology assessment, and motif visualization. The IDs were manually investigated to assess protein location, potential function, homology to proteins in other species, and the presence of variant transcripts. Function was assessed partially through domain descriptions available through the Pfam database (Finn et al., 2015), allowing for inferences about domain activity. Genomes investigated for homology include: *Aegilops tauschii*, *Amborella trichopoda*, *Arabidopsis thaliana*, *Brachypodium distachyon*, *Hordeum vulgare*, *Musa acuminata*, *Oryza sativa*, *Setaria italica*, *Triticum urartu*, and *Zea mays*. Genomic data was not available for *Aegilops speltoides*, which is believed to be the contributor of wheat’s B genome. Special attention was paid to the relationship between wheat NLRs and their homologs in the two progenitors of wheat: *Triticum urartu* and *Aegilops tauschii*. These results were visualized to show how many NLR-ID fusions are shared between wheat and its progenitors in order to assess how many fusions took place before their divergence and how many may have happened since their divergence.

Alternative transcripts, also downloaded from the databases mentioned above, were assessed for ID motifs. Among the NLR-ID accessions, genes were identified for the transcripts where IDs were present in some but absent in others. The Gene Structure Display Server 2.0 (Hu et al., 2014) was used to visualize alternative splicing of NLR-IDs. Wheat expression data was acquired from the NCBI database and Wheat Gene Expression Atlas data (Borrill et al., 2016). The alternative transcript data was mapped onto expression data, showing experimental evidence that these alternative transcripts were expressed in wheat tissue.

### Plasmid Constructions

Six genomic fragments of *OsRPR1* were PCR-amplified from *Oryza sativa* Japonica genomic DNA with primers (Table 1) containing 4bp specific overhangs and *BsaI* recognition sequence. The amplified products were cloned into the pCRTM8/GW/TOPO (Invitrogen K2500-20) vector. The resulting constructs, together with a *C*-terminal YFP tag, were subsequently used for Golden Gate assembly in pICH86988 (a kind gift from Dr. Sylvestre Marillonnet), thus generating the pICH86988::OsRPR1:YFP construct. For cloning of *OsRPR1* CDS, the amplified PCR product of *OsRPR1* (see below) was cloned into pICSL01005.

**TABLE 1:** Primers used for *OsRPR1* (OsJ_34782) cloning.

Name	Sequence
OSRPR1-FRG1 FW	TTGGTCTCCAATGTTCAACCTCCCCAGGAG
OSRPR1-FRG1 RV	TTGGTCTCACTATTCTGCGATCTGGCGGCATGCTC
OSRPR1-FRG2 FW	TTGGTCTCAATAGACCTAATATTCACCACCGTAAG
OSRPR1-FRG2 RV	TTGGTCTCAGTGCCTGCTAGAATTTGCATGATG
OSRPR1-FRG3 FW	TTGGTCTCAGCACTAACAATAATTAGAGCTTCC
OSRPR1-FRG3 RV	TTGGTCTCAAGTGAAAGTTTTCTTATTTCTGACAC
OSRPR1-FRG4 FW	TTGGTCTCACACTAGATGTGCACATACACATCTCAAG
OSRPR1-FRG4 RV	TTGGTCTCACTCCTCATGGAGCTCCCCACTAC
OSRPR1-FRG5 FW	TGGTCTCAGGAGTGGTGAGTGCCAGGT
OSRPR1-FRG5 RV	CCGCGGTCTCACTTGAAATCAATTTTATAACAC
OSRPR1-FRG6 FW	TTGGTCTCACAAGTCTACCATGCACGCAC
OSRPR1-FRG6 RV	TGGTCTCTCGAACCACCAGTAACCCAATCACAGCCC
OSRPR1-SV-FW	GGAGGATGACGGCCTCAG
OSRPR1-SV-RV	CCAGTAACCCAATCACAGCC

### Agrobacterium-Mediated Transient Expression in *Nicotiana benthamiana*

For agroinfiltration in *Nicotiana benthamiana, Agrobacterium tumefaciens* strain AGL1 was transformed with the binary constructs by electroporation. *Agrobacterium* strains carrying the construct were grown in 5 ml liquid LB-medium supplemented with adequate antibiotic for 24 h. Cells were harvested by centrifugation, washed twice in 10 ml of 10 mM MgCl_2_ and re-suspended at OD_600_ 0.5 in infiltration medium (10 mM MgCl_2_, 10 mM MES pH 5.6). Agroinfiltration was performed with 1 ml needleless syringe in 4–5 week-old *N. benthamiana* leaves.

### RNA Extraction and RT-PCR

Total RNA was isolated from deep-frozen plant material using the TRIzol^®^ method (Invitrogen) according to the manufacturer’s specifications. For cDNA synthesis, 1 μg of DNaseI (NEB) treated total RNA was reverse transcribed using Superscript II (Invitrogen). For the amplification of *OsRPR1* CDS, PCR was conducted with Phusion Polymerase (NEB) using the primers OsRPR1-Frg1 Fw and Frg5 Rv (Table 1) for 35 cycles following manufacturer’s instructions. The correct size band was harvested from the gel. For the identification of other spliceforms at the 3′ end of *OsRPR1* CDS, PCR was performed using Phusion Polymerase (NEB) with the primer set OsRPR1-SV-Fw and OsRPR1-SV-Rv (Table 1) for 35 cycles following manufacturer’s instructions.

## Results

### Wheat NB-ARC-Encoding Proteins

**FIGURE 1:**
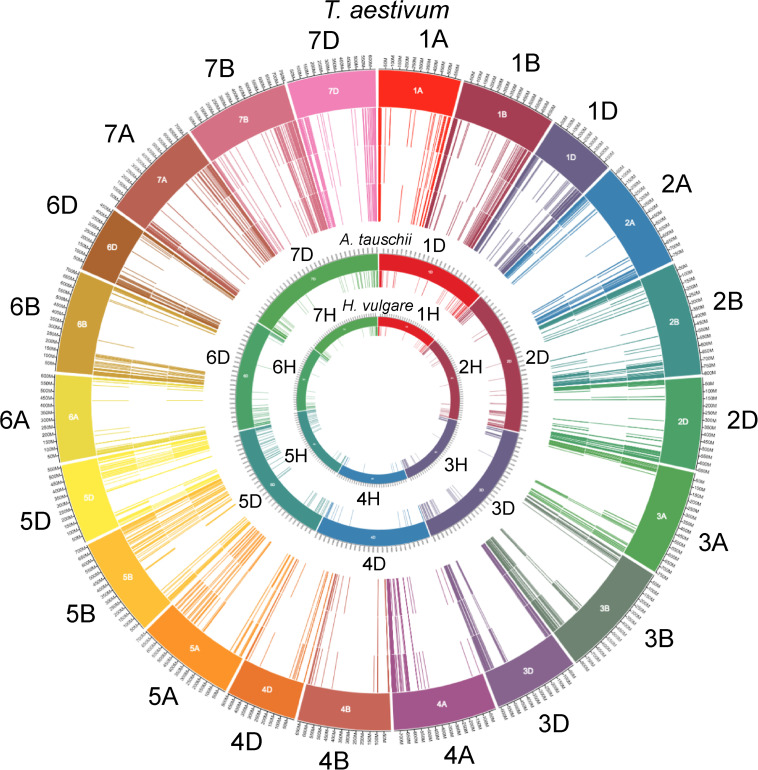
NB-ARC-encoding gene locations on the chromosomes of wheat, Aegilops and barley. The outermost track of lines indicates accession names of *T. aestivum* NB-ARC encoding genes on the chromosomal arms (1–7; A, B, and D; represented in the outermost circle of color-coded blocks). Inner next three circles of lines from outside to inside represent total number of NB-ARC encoding genes, genes encoding CC domain and those with the LRR domains, respectively. Inner to the *T. aestivum* circle, are the locations (chromosome 1–7D color coded) of the NB-ARC-encoding genes in *Aegilops tauschii* (contributor of the wheat D sub-genome). The locations of all NB-ARC encoding genes (close relative of the wheat progenitors) in the genome of *Hordeum vulgar*e are represented in the innermost circle. To avoid the cluttering, circles of the sub-classes of the NB-ARC encoding genes of the latter two species are not included. For all 2,151 NB-ARC gene accessions and their major categories, see Supplementary File 1.

**FIGURE 2:**
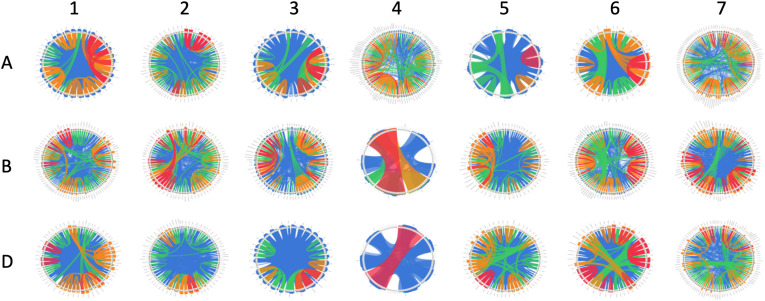
Similarity of clustered NB-ARC-encoding genes on each chromosome. Chromosomes are shown 1–7 with sub-genomes **(A–D)** from superior to inferior. Clustered genes are arranged in a circle, with lines describing <50% similarity, <75% similarity, <99.9999% similarity, and 100% similarity in blue, green, orange, and red, respectively.

The wheat genome contains many genes that encode for NB-ARCs. Approximately half of wheat’s 2,151 NB-ARC-encoding proteins also contained a CC domain, and approximately 75% encoded LRRs (Figure 1). Accessions and sequence data for Figure 1 are given in Supplementary File 1. Of the 2,151 NB-ARC-encoding genes, 1,505 had NB-ARCs with P-loop, kinase-2, and GLPL motifs. Among the 1,552 proteins with the LRRs, 802 contained coiled-coil (CC) domains (CNL) and three had resistance to powdery mildew 8 (RPW8) domains (RNL). Interestingly, five of the NB-ARC-encoding genes encoded a toll/interleukin-1 receptor (TIR) with no LRR (TN proteins). NB-ARC-encoding genes formed 547 gene clusters; highly similar clusters are visualized in Figure 2. Many of the clustered genes possessed greater than 75% similarity within each cluster. While chromosomes from each of the wheat sub-genomes are very similar (e.g., chromosomes 1A, 1B, and 1D), differences emerged between the gene clusters found on each chromosome. For example, in wheat’s fourth homologous chromosome group (4A, 4B, and 4D), chromosome 4A contained 57 clusters (involving 119 genes), 4B contained four clusters (involving eight genes), and 4D contained three clusters (involving seven genes). It is unknown whether this diversification in 4A happened prior to the first or second hybridization event in wheat. Since *Triticum urartu* gene locations were not available, a clear understanding of the difference between *T. urartu* chromosome 4 and wheat chromosome 4A could not be established.

### Integrated Domains in Wheat

**FIGURE 3:**
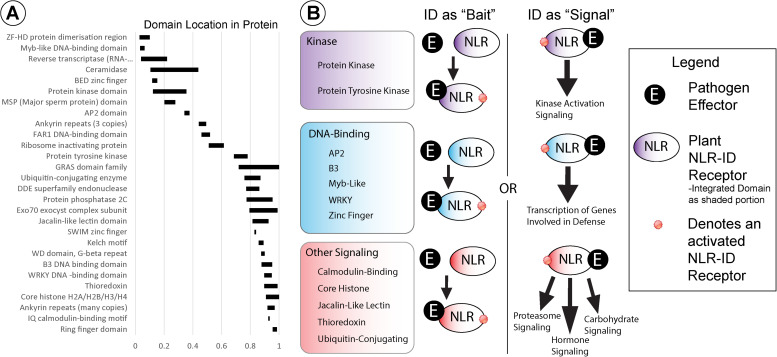
**(A)** Integrated domain (ID) locations, indicated by black rectangles, are shown within NLRs relative to protein length (0–1). **(B)** IDs were grouped into functional categories, based on their potential involvement in kinase, DNA-binding, or other signaling activities (shown in purple, blue, and red, respectively). Schematic diagrams representing potential functions for these NLR-IDs are included with pathogenic effectors represented by black circles and NLR-ID proteins as ovals color coded by ID type (i.e., “kinase,” “DNA-binding,” or “other signaling”), as shown in the figure legend. The diagram includes representations of the kinds of effector-bait interactions and NLR-ID signaling pathways in which these domains may be involved.

Several wheat NB-ARC-encoding genes also encode for IDs that may function as molecular baits, decoys, or signal transduction factors. Wheat NLRs possess a diverse set of IDs, the most common of which are kinase and DNA-binding domains. Figure 3 shows the average location of 28 different types of IDs relative to protein length, with averages calculated from every NLR-ID occurrence of that domain. Kinase domains are generally located in the *N*-terminal half of the protein, and tyrosine kinase domains are generally in the middle of the protein sequence. DNA-binding domains, which are more diverse (e.g., AP2, BED zinc finger, Myb-like, and WKRY), vary by domain type and may be present in either the *N*- or *C*-terminus. For example, Myb-like and BED zinc finger domains are generally located at the *N*-terminus, while B3 and WRKY domains are located at the *C*-terminus. Many other IDs associated with the *C*-terminus, including calmodulin-binding, jacalin-like lectin, thioredoxin, and ubiquitin-conjugating domains, may have roles in signaling.

### Integrated Domain Homology

**FIGURE 4:**
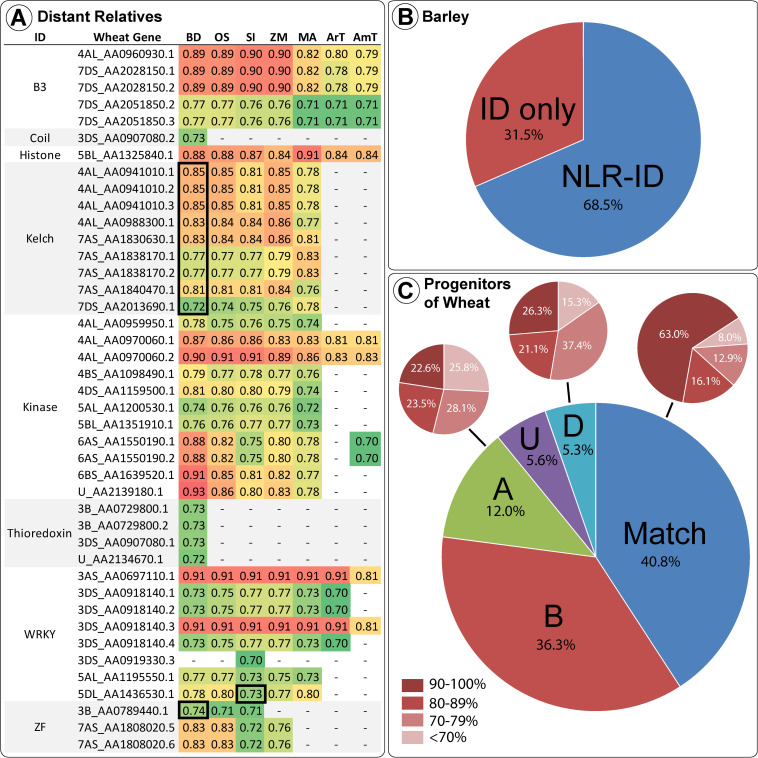
Wheat IDs and their homologs in wheat progenitors and other divergent monocot species are shown, including *Arabidopsis thaliana* and *Amborella trichopoda.* **(A)** Sequence similarities above 70% are shown between wheat IDs and their homologs in Brachypodium (BD), rice (OS), foxtail millet (SI), maize (ZM), banana (MA), *Arabidopsis* (ArT), and *Amborella* (AmT). Wheat gene accession names are abbreviated to include information on chromosome arm and the last digits unique to each transcript. Sequence similarities of the NLR-ID homologs in other species are shown in the black-bordered boxes. **(B)** Barley ID homologs possessing and lacking NLR domains. **(C)** Mapping of homologs among wheat and wheat progenitors is displayed – a match between the progenitor and subgenome (labeled “Match”); subgenome A protein was more similar to an *Aegilops tauschii* sequence (labeled “A”); subgenome D protein was more similar to a TU sequence (labeled “D”); sequence was from the B subgenome with the unavailable AS progenitor (labeled “B”), or the accession subgenome is unknown (labeled “U”). Also, the level of homology between the pairs is demonstrated for “Match,” “A,” and “D,” with dark red corresponding to the proportion of sequences with high similarity (>90%) and lighter red corresponding to lower similarity (<70%).

Many wheat IDs share homology with proteins in distantly related monocots. Figure 4 shows the wheat accessions with high percent identity (above 70%) grouped by ID type and homolog species. The vast majority of these ID homologs in other species do not contain NB-ARC domains, as shown by the minority of homology scores surrounded by thick black lines in Figure 4. This lack of NB-ARCs in homologs indicates recent fusions that took place after the species diverged or loss in related species, where the few NLR-ID homologs, present mostly in *Brachypodium distachyon*, indicate an ancient fusion that took place before the species diverged. ID homologs in more distant relatives were not NLR proteins, such as the homologs in *Arabidopsis thaliana* and *Amborella trichopoda*. While other plants also possess NLR-ID fusions, many are lineage specific and are not conserved across diverse species. Barley, a close relative of wheat, possessed many of the same NLR-ID fusion proteins as wheat, with 68.5% of ID homologs in barley also possessing NLRs. The two progenitors of wheat with sequenced genomes, *T. urartu* and *A. tauschii*, also possess wheat’s NLR-ID fusions. Of these progenitor homologs, 40.8% matched the expected subgenome according to the known progenitor-subgenome relationships. Genomes investigated for homology include: *Aegilops tauschii* (AT), *Amborella trichopoda* (AmT), *Arabidopsis thaliana* (ArT), *Brachypodium distachyon* (BD), *Hordeum vulgare* (HV), *Musa acuminata* (MA), *Oryza sativa* (OS), *Setaria italica* (SI), *Triticum urartu* (TU), and *Zea mays* (ZM). Genomic data was not available for *Aegilops speltoides* (AS).

### NLR-ID Regulation

**FIGURE 5:**
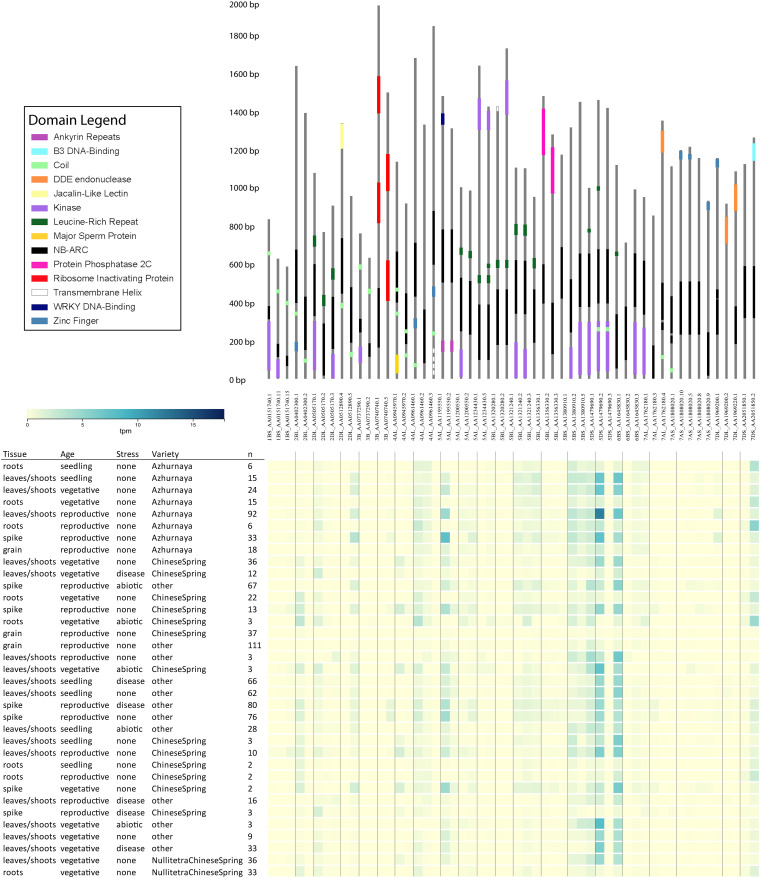
Alternative transcripts of wheat NLR-ID genes in which IDs were excluded or truncated in at least one alternative transcript. Upper panel shows the gene models of the NLR-ID genes. Domains are color-coded as defined in the domain legend. Wheat accession names include information on chromosome arm, accession number, and transcript number. Below the gene models are expression data from the Wheat Gene Expression Atlas, showing that these alternative transcripts can be experimentally tested for their expression in wheat tissues. The expression of these transcripts varies by variety, type of stress, and tissue type. Visualization layout was made based upon expVIP within the Wheat Gene Expression Atlas database.

Some wheat NLR-ID genes encode alternative transcripts that omit IDs or domains associated with traditionally understood NLR function. Figure 5 illustrates a consolidation of all wheat NLR-IDs in which at least one alternative transcript of the gene excluded the ID. Alternative splicing of this kind would allow plants to regulate the use of IDs by including or excluding exons containing them. Similar characteristics were also observed in barley transcripts, indicating a conserved use of alternative splicing. Alternative transcripts may also be found in wheat progenitors, which currently lack available data. Expression data from the Wheat Gene Expression Atlas and NCBI shows differential expression between these alternative transcripts, which are also shown in Figure 5. This expression data verifies that these alternative transcripts are actually expressed, and that many of them are expressed at different rates depending upon experimental conditions.

### Intron Retention in the Coding Sequence of OsRPR1

**FIGURE 6:**
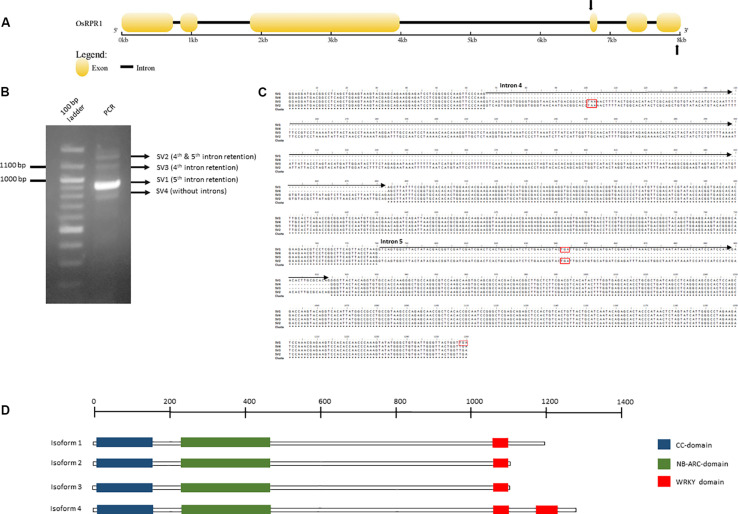
Organization of the *OsRPR1* gene **(A)** and the PCR amplified splice variants produced after PCR amplification with primers spanning the fourth and sixth exon **(B**, **C)**. The putative isoforms rising from this splice variants and their domains are depicted in **(D)**. The complete DNA sequences of the putative splice variants and the corresponding isoforms are provided in Supplementary File 2.

Alternative splicing of NLR-IDs was investigated in another monocot, *O. sativa*. We identified *in silico* an NLR-ID gene (Figure 6), which we termed *Resistance Paired Receptor 1* (*OsRPR1*) (Locus EEE52547.1; Os11g45750) that contains two WRKY domains at its *C*-terminus. The gene was cloned into a plant expression vector and overexpressed in *N. benthamiana* leaves. The coding sequence of the gene, which was amplified from cDNA generated from total RNA from the agroinfiltrated area, was cloned and sequenced. We found that exon 5 was retained in the coding sequence of *OsRPR1*. In order to investigate whether other splice variants rise from the same *C*-terminal region of the gene, we performed a PCR with primers spanning this region (Figure 6A). Three out of the four amplified bands were sequenced (SV1, SV2, and SV4; see whole sequences in Supplementary File 2). As expected, the most intense band corresponded to the splice variant of *OsRPR1* which retains the 5th exon (SV1). The three other bands corresponded to splice variants containing the 4th and 5th introns (SV2), the 4th intron (SV3), or no introns (SV4) (Figures 6B,C). These splice variants have either longer 3′ UTRs (SV1, SV2, and SV3 vs SV4) or probably code for different isoforms (SV1 and SV4 vs SV2 and SV3).

## Discussion

### Wheat NB-ARC-Encoding Genes

Nucleotide-binding leucine-rich repeat receptor systems use groups of sensor and helper NLRs to detect and initiate defense responses when pathogenic effectors are present (Jones et al., 2016). Not all functional NLRs have all characteristic domains (TIR/CC and LRR), such as Pb1 (Hayashi et al., 2010), and some have extra domains (Bailey et al., 2018). Therefore, proteins lacking CC or LRR domains may also contribute to resistance responses, especially those with additional domains that are involved in signaling (Baggs et al., 2017). The distribution of NB-ARC-encoding genes across wheat chromosomes concurs with previous studies in barley and foxtail millet, where R-genes were also found in clusters in extra-pericentromeric regions of chromosomes (Andersen et al., 2016; Andersen and Nepal, 2017). Unequal crossing over between chromosomes as a mechanism for duplication likely explains the formation of these clusters. Previous studies have highlighted this explanation for the locations of the quickly evolving genes (Marone et al., 2013). *A. tauschii* and *H. vulgare* share a similar pattern as wheat (Figure 1), with a similar number of R-genes located at the ends of chromosomes. Barley and the progenitors of wheat diverged approximately 8–9 million years ago (Middleton et al., 2014). Both barley and wheat have experienced artificial selection, as both have been grown for food production since the agricultural revolution approximately 10,000 years ago. Wheat differs from barley in that it is an allohexaploid resulting from hybridization of three species, each containing seven pairs of chromosomes, while barley remained diploid with only seven pairs of chromosomes. Wheat and *A. tauschii* differ in that *A. tauschii* did not experience selective breeding like wheat did. The wheat genome, consisting of A, B, and D subgenomes, maps to the barley genome (H), with wheat chromosomes 1A, 1B, and 1D containing much synteny to 1H of barley. Instances exist where barley possesses duplicated genes that remained unduplicated in wheat, and vice versa. The similarity between wheat and *A. tauschii* is much closer, since *A. tauschii* contributed wheat’s D subgenome only a few thousand years ago. While the A subgenome progenitor, *Triticum urartu*, has limited genomic availability, future studies may be able to assess differences between NLR gene architecture in the two genomes. *A. tauschii* and barley provide excellent comparisons with wheat due to the relatively short period of time since their divergence. The similarities between R-genes in wheat relatives show that the highly diverse family of R-genes is necessary for survival, whereas the differences in number and phylogeny point to differences in selection pressure that these species each face.

Clustered genes tend to contain highly similar sequences, indicating that they result from tandem duplication. Some genes, for example, were separated by only a few hundred nucleotides and shared >90% similarity (Figure 2). Through tandem duplication, wheat NLR genes may have diversified to respond to rapidly evolving and perhaps closely related pathogens, such as those with complex pathotype or race structures, like *Pyrenophora tritici-repentis* (Abdullah, 2017). Race-specific (vertical) resistance in wheat has been identified with regard to pathogens such as powdery mildew (Bourras et al., 2015). Horizontal resistance may include other signaling factors and types of receptors and may rely only partially on NLRs (Kushalappa et al., 2016). While many clustered genes were similar, several cases were identified in which genes were located close to one another but were dissimilar and did not nest together in a phylogenetic analysis. This phenomenon has two major explanations: (1) the tandem duplication took place long ago in evolutionary history and these genes had time to substantially diversify, or (2) a segmental duplication took place, causing the gene to become located next to another R-gene or R-gene cluster. R-genes are highly diversified in plants, with many species possessing hundreds of them. Ancient tandem duplications would have had time to diversify, especially if selective pressures acted upon the ancestors of modern species. However, segmental duplications cannot be discounted due to the presence of genes in some clusters that are highly similar to genes in other clusters (Leister, 2004). In these cases, transposable elements may play some role in the movement of these genes among different chromosomes or to distant locations on the same chromosome (Kim et al., 2017).

### Integrated Domains May Augment NLR Function Through Signaling and Recognition

Kinase and DNA-binding IDs likely function as signaling domains that help NLRs initiate defense responses. Current models of NLR function describe a conformational shift triggered when pathogenic effectors bind to the *C*-terminal LRR, causing the NB-ARC to exchange ADP for ATP and opening the protein up for the N-terminus to initiate further signaling (Takken and Goverse, 2012; Michelmore et al., 2013; Cui et al., 2015). LRRs, as highly variable domains of repeating Lxx amino acid residues, allow defense receptors to bind to diverse elicitors. The NB-ARC, as a P-loop-containing nucleoside triphosphate hydrolase, functions in hydrolysis of beta-gamma phosphate bonds in ATP, binding to phosphates using the Walker A (P-loop) motif and to magnesium ions necessary for catalysis by Walker B motifs (Walker et al., 1982). This release of energy from ATP hydrolysis drives protein conformational change, allowing *N*-terminal domains (i.e., TIR or CC) to trigger downstream signaling. Kinase IDs found in wheat NLRs could initiate signaling through phosphorylation of transcription factors or other kinases (i.e., MAPK). Sarris et al. (2016) also found an abundance of NLR-kinase fusions, which possibly retain their biochemical activity (Sarris et al., 2016). DNA-binding domains could move directly to the nucleus upon activation, binding to promoters of pathogenesis-related (PR) genes to recruit transcription machinery. IDs that likely bind to DNA include: AP2, B3, zinc finger, Myb, and WRKY domains, which have been shown to play roles in pathogen resistance (Gutterson and Reuber, 2004; Buscaill and Rivas, 2014). The *Arabidopsis* NLR gene AT4G12020 has been identified both as MAPKKK11 (Jonak et al., 2002) and a TNL resistance gene (Meyers et al., 2003), containing WRKY DNA-binding sites and a protein kinase domain. This gene is a homolog of SLH1, which has been associated with hypersensitive response and may function as a guard for a pathogen effector target (Noutoshi et al., 2005). Many NLR-ID fusion proteins contain transmembrane (TM) domains or nuclear localization signals (NLSs). Several proteins have multiple transmembrane domains, with proteins like 3B_AA0787000 containing seven that are characteristic of other transmembrane proteins. NLSs indicate that DNA-binding domains may functionally interact with DNA as transcription factors.

In addition to signaling, some IDs may play direct roles in effector recognition as effector-binding domains or bait domains that mimic effector targets (Figure 3). Jacalin-like lectin domains, for example, bind to carbohydrates and can recognize carbohydrates that originate directly from pathogens or from damage incurred during infection (Xiang et al., 2011; Lannoo and Van Damme, 2014; Esch and Schaffrath, 2017). Mannose-binding lectin domains were also found in NLRs, associated with disease resistance (Hwang and Hwang, 2011), along with “wall-associated receptor kinase galacturonan-binding” and “cleavage site for pathogenic type III effector avirulence factor Avr” domains. Lectin domains may distinguish proteins as helper NLRs, with carbohydrates acting as signals to initiate NLR activation. Other domains may play roles in effector recognition as bait domains that resemble effector targets. The resistance protein RRS1 becomes activated when an integrated WRKY domain interacts with *Ralstonia solanacearum* effector PopP2 and *Pseudomonas syringae* pv. *pisi* effector AvrRps4, effectors that otherwise target WRKY transcription factors (Le Roux et al., 2015; Sarris et al., 2015). Wheat NLR-WRKY fusions share homology with WRKY16, WRKY19, WRKY46, and WRKY54/70, with potential roles as targets, especially WRKY46, which is associated with bacterial resistance (Sarris et al., 2016). Variants of WRKY domains (WRKY and WSKY) were found and may provide diverse baits for effectors. Genes also encode for multiple transcription factor IDs, which may allow proteins to bind to separate promoters or act as bait for multiple effectors. Some bait proteins, such as PBS1, are kinases that pathogen effectors target for degradation, and may thus increase the utility of NLR-kinase fusions. The Rosetta stone theory posits that such associations between fused domains may indicate functional interactions (Date, 2008). Several proteins with IDs and NB-ARCs do not contain LRRs, which would not be required for activation since baits have replaced LRRs in function.

The activity of IDs as baits is further supported by ID diversity, which corresponds to the diversity of defense regulatory components. IDs found in NLRs are also found in proteins that effectors target to interfere with defense. Several domains correspond to proteins involved in resistance signaling: calmodulin-binding (calcium signaling), Gibberellic acid insensitive (GAI) repressor of GAI and scarecrow (GRAS; gibberellin signaling), and ethylene responsive element binding (ethylene signaling). Several different domains contain IDs associated with the proteasome or ubiquitin, and these include protease subunit, proteasome component signature, cullin-repeat, RING/U-box, ubiquitin conjugating enzyme, and WD domains. Some IDs contain domains associated with regulation of DNA expression: core histone and chromatin organization modifier. Other IDs correspond to proteins involved in resistance responses: ribosome inactivating and ricin domains (disrupt ribosome activity), thioredoxin and kelch (oxidase activity in reactive oxygen species production), alpha subunit of tryptophan synthesis (synthesis of antiherbivory and antimicrobial compounds), Exo70 exocyst complex subunit (transport of antimicrobial compounds out of the cell), and DDE endonuclease (apoptosis). A few other domains are likely associated with pathogen components: major sperm protein (nematode sperm function, targeted by plant RNA interference), FNIP (found in *Dictyostelium discoideum*), and reverse transcriptase (inhibition of viral infection). Additional viral IDs include RNA-binding/recognition, retrovirus zinc finger-like domain, and integrase domains. IDs may also be associated with pathogen-derived resistance and RNAi that plants use to inhibit viruses and other pathogens. Other studies have found a similar degree of ID diversity in other plant species (Sarris et al., 2016; Baggs et al., 2017).

Our results concur with those presented in Bailey et al. (2018), were a similar set of NLR-IDs were found in the wheat genome, including kinases and transcription factors, of which AP2 was a focus of their study. Bailey et al. (2018) found that certain phylogenetic clades of NLRs contained disproportionately high contents of IDs, which were called major integration clades (MICs), which 30% of NLR-IDs belonged. Many of these were complete domains, consistent with our results. Bailey et al. (2018) proposed retrotransposition, transposition, and ectopic recombination as potential mechanisms for NLR-ID formation. A significant amount (approximately 10%) of NLRs contain exogenous domains, consistent with these results (Kroj et al., 2016; Sarris et al., 2016; Bailey et al., 2018). Our results show that IDs are diverse, but kinases and transcription factor IDs are very common and may due to the presence of MICs where several closely related genes share similar IDs.

### Integrated Domains Evolve as Functional Domains Shared by Close Relatives

As a monocot, wheat shares distant relationships with other members of the family Poaceae (i.e., BD, ZM, SI, and OS). ID homologs in distant relatives generally do not contain NB-ARCs, indicating relatively recent origin of NLR-ID fusions. IDs with high percent similarity to homologs (Figure 4) may indicate functional retention. These include proteasome subunit, B3 DNA-binding, WRKY DNA-binding, core histone, protein kinase, and kelch motif. Other domains with moderate similarity include: jacalin-like lectins, ribosome-inactivating protein, BED zinc finger, SWIM zinc finger, ZF-HD protein dimerization region, zinc knuckle, protein phosphatase 2C, tyrosine kinase, thioredoxin, major sperm protein, reverse transcriptase, and DDE endonuclease. Homologs may be obscured, since mutations accumulate in regions not essential for function or effector-bait interaction. This would cause divergence from the original sequences and would make homology difficult to assess. Some mutations may increase the functionality of NLR-IDs, since the original ID sequence was functionally optimized within a different protein. Some IDs that possess similar modification/cleavage sites may serve as baits for multiple targets (e.g., similar WRKY domains). Many IDs showed high homology in distant relatives. Kinase domains of up to 300 amino acids in length were over 80% similar to homologs. DNA-binding domains also had high homology in distant relatives. WRKY DNA binding domains present in wheat and progenitors have 90.5% similarity to several non-NLR genes in AT, BD, MA, OS, SI, and ZM. Many other IDs in wheat and its progenitors share >80% similarity with homologs in SI, ZM, BD, OS, AT, MA, and AmT. These results concur with previous investigations into IDs, where conserved IDs were identified in diverse plant species (Kroj et al., 2016; Sarris et al., 2016). Some wheat proteins are very similar to their homologs in TU and AT, whereas others provide examples of proteins in one species diversifying from the other two. The histone ID in wheat protein 5BL_AA1325840 (approximately 100 amino acids) shares strong homology (>80%) with proteins in MA, BD, OS, SI, ArT, AmT, and ZM, a recent fusion not present in wheat relatives. Greater than 90% similarity was observed between the 182 amino acid long F775_12304| EMT01588 proteasome subunit domain and proteins in BD, OS, SI, and ZM. While this indicates that these accessions are close homologs, none of the other accessions have NB-ARCs and instead include only peptidase, proteasome subunit, and nucleophile aminohydrolase domains. HV, TU, and TA homologs to this domain, while matching the sequence 100%, do not have NB-ARCs, indicating a very recent duplication and then fusion that occurred after the hybridization of hexaploid wheat. Kelch motif IDs were found in one TU, three AT, and six TA proteins. Interestingly, only one of the TA proteins is in the D sub-genome, and five were located in the A sub-genome, while sub-genome origin would suggest that the opposite pattern would be expected. Bailey et al. (2018) also found that many IDs in wheat’s A and D sub-genomes correspond to IDs in TU and TA, with additional IDs that resulted after wheat split from its progenitors.

While domain homology in distantly related species indicates functional origins of IDs, homologs identified in close relatives (i.e., HV) and wheat progenitors (TU and AT) indicate recent fusions and duplications. Unlike distant relatives, barley shares many NLR-ID fusions with wheat. This indicates that many of wheat’s NLR-IDs happened before the divergence of barley and wheat progenitors. Since this divergence, wheat and barley have independently gained and lost NLR-ID proteins. Many TU and AT proteins are almost identical to proteins encoded by TA genes within the A and D sub-genomes. IDs within wheat’s B sub-genome often originate from AS and do not have 100% homologs in TU and AT. In select cases, similarity was found between NLR-IDs and functional domains from non-NLR proteins, indicating potential NLR-ID fusions since the formation of wheat. Conversely, some close wheat relatives share homology with distantly related NLR-ID fusions, such as F775_00546| EMT17242 and Si008625m, with an 84.6% similarity (991 identical sites) between their whole sequences, both with NB-ARCs and kinase domains. Unexpectedly, many proteins were found in wheat but not a relative, or vice versa. These domains include histone, ribosome inactivating protein, calcium signaling, cleavage for type III effectors, RNase H type, P450, antibiotic synthesis, and ubiquitin conjugating enzyme. Other domains were found in greater or lower numbers in wheat compared to its progenitors, such as DDE endonuclease and reverse transcriptase, indicating loss or duplication in one genome.

### Plants Use Alternative Splicing to Regulate NLR-IDs

**FIGURE 7:**
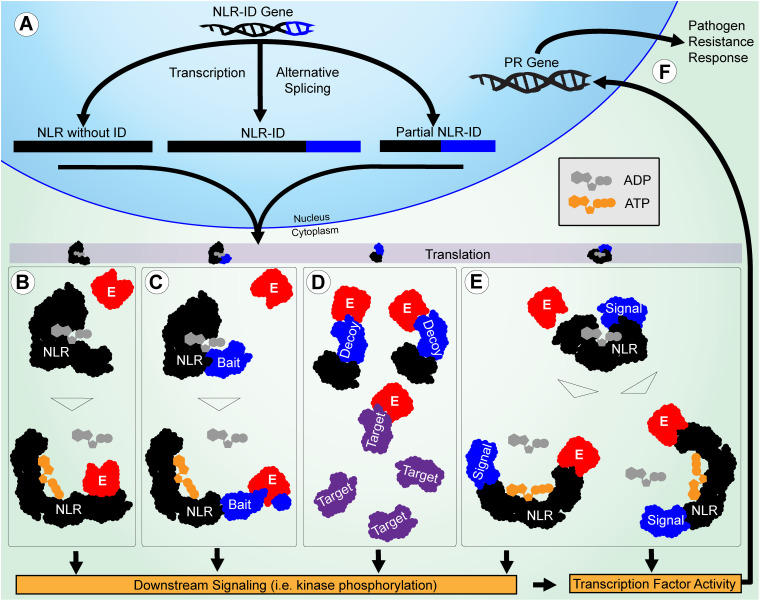
Potential roles of IDs in functional diversification of NLRs in pathogen resistance are illustrated. **(A)** The NLR-ID gene is alternatively spliced during transcription to include or exclude IDs. The NLR and ID sequences are shown in black and blue, respectively. **(B)** The NLRs without IDs function through effector-LRR binding to activate the protein and trigger downstream signaling. Effectors are shown in red. **(C)** When IDs are used as baits, they mimic pathogen targets and cause NLR activation after they are modified. **(D)** When IDs are used as decoys, they mimic pathogen targets to reduce effector interference in resistance signaling. The targets are shown in purple. **(E)** When IDs are used in signaling, they allow NLRs to act as signal transduction factors, making them less reliant on the downstream signaling utilized by other NLRs. **(F)** Finally, transcription factor activity directly involving or triggered by NLRs causes PR genes to be expressed, leading to a resistance response.

Many NLR-ID protein-encoding genes possess multiple transcripts, some of which lack IDs or truncate domains within the protein (Figure 5). This indicates that plants may use alternative splicing to regulate the use of IDs within a network of NLR proteins. Previous studies have shown that resistance to some pathogens requires alternative splicing (Yang et al., 2014; Shang et al., 2017), such as *RPS4* in *Arabidopsis* (Zhang and Gassmann, 2003); and splicing is used to truncate proteins like *RCT1* in *Medicago truncatula* (Tang et al., 2013). Wheat has also shown evidence of alternative splicing of important resistance genes Lr10 and Sr35 (Sela et al., 2012; Saintenac et al., 2013). Splicing patterns between wheat paralogs resulting from duplication also appear to be conserved. Stop codon-containing inter-exon regions can be included in the transcript to force a truncation of the protein. The results presented in Figure 6 show that R-genes encode splice variants, and that introns were retained in the coding sequence, indicating further R gene variation is possible through alternative splicing. Truncated NB-ARCs may result in decoy proteins, where signaling function is lost but IDs ‘distract’ pathogenic effectors from functional target proteins. The potential involvement of alternative splicing is shown in Figure 7. In concurrence with the results, Yang et al. (2014) described NLR alternative splicing as useful for regulating NLR autoinhibition or function in signal transduction and also detailed potential transcription factor activity (Yang et al., 2014). Genes that have multiple copies of an ID can also regulate the number of copies in the protein through alternative splicing. Alternative splicing may also allow the plant to select different localization for a gene product, such as those where transmembrane helices are included in one transcript and are not included in another (Figure 5).

Wheat expression data shows that there are differences in the expression of these alternative transcripts shown in Figure 5. The expression values for the 54 transcripts present in Figure 5 were mined from datasets present in the Wheat Gene Expression Atlas and NCBI databases. Expression data from Salcedo et al. (2017) shows that alternative splicing may result from different conditions (Salcedo et al., 2017). At the very least, these data provide support for Figure 5 accessions as confirmed alternative transcripts that differ in expression. In the Wheat Gene Expression Atlas data (Borrill et al., 2016), several transcripts with different ID contents show differential expression in wheat tissues. Several genes were more strongly expressed in the leaves, shoots, and spikes, indicating the potential tissue-specific roles that these genes play in resistance. More data is required to conclusively show differential expression based upon certain treatments and conditions. While wheat shows evidence of NLR-ID alternative splicing, barley may have evolved a more diverse set of transcripts for NLR-IDs. Several barley NLR-ID proteins have dozens of transcripts, with several of those allowing for alternative uses of IDs in NLR proteins. Many barley genes have alternative transcripts that encode NLR-ID, just NLR, just ID, or lack both. Previous studies have identified barley *Mla* genes as utilizing alternative splicing for resistance (Halterman et al., 2003). Barley genes can also encode multiple IDs. Barley data also supports a previous prediction that alternative splicing may allow for differential cellular localization (Yang et al., 2014).

## Conclusion

In this study, we identified 2,151 NB-ARC-encoding genes in the wheat genome, with many encoding additional domains associated with the receptors that detect pathogenic effectors. In the 21 chromosomes of wheat, 547 gene clusters were found, with many clusters containing highly similar genes. Clustering of R-genes in wheat was compared to its progenitors and barley, a close relative, and gene similarities within clusters showed that tandem duplication explains much of the diversification among R-genes. The diversity of IDs in NLRs corresponds directly to the multiple components utilized by plant cells to initiate resistance responses. These components included kinases, transcription factors, hormone signaling receptors, and proteins involved in antimicrobial compound production. NLR-ID fusions give these immune receptors the potential to function as effector baits, decoys, and signal transduction factors. Sequence homology indicates that some IDs may retain functionality and evolve into non-NLR proteins. Genomic and gene expression data suggest that plants likely utilize alternative splicing to regulate the inclusion or exclusion of IDs in NLR proteins. We tested it in rice, another Poaceae member, where Agrobacterium-mediated transformation of *OsRPR1* gene exhibited alternative splicing. The ability of plants to use splicing to include or exclude IDs constitutes an important defense strategy to deal with rapidly evolving pathogen effectors. Future studies should aim to characterize the structure of NLR-ID fusion proteins, demonstrate which IDs have retained enzymatic activity, and associate the expression of alternative transcripts with specific conditions. Additional genomic data on *Aegilops speltoides*, a relative to the contributor of wheat’s B sub-genome, as well as availability of data for *Triticum urartu*, contributor of wheat’s A sub-genome, will allow for more thorough analyses of the evolution of disease resistance genes in wheat.

## Data Availability Statement

The datasets generated for this study can be found in the https://figshare.com/articles/Table_S1_Expression_of_wheat_NLR-ID_alternative_splicing_candidate_genes_available_in_Wheat_Gene_Expression_Atlas_and_NCBI_databases_/8796449.

## Author Contributions

EA and GM carried out the experiment and analyzed the sequence data. MN and PS conceived the research project and supervised the experiment. JP and DN contributed drafting the manuscript. All authors read and approved the final manuscript.

## Conflict of Interest

The authors declare that the research was conducted in the absence of any commercial or financial relationships that could be construed as a potential conflict of interest.

## Footnotes

1 http://omgenomics.com/circa

## Supplementary Material

FILES 1 Wheat accession, sequence, and location data for the genes shown in Figure 1. Table can be accessed at the Figshare link: https://figshare.com/s/78d566a9c4cf4902d283.

FILES 2 Genomic sequences of the splice variants and the corresponding isoforms shown in Figure 6.
