# Supplementary material for: Wheat Disease Resistance Genes and Their Diversification Through Integrated Domain Fusions
Source: Front Genet. 2020 Aug 5;11:898. doi: 10.3389/fgene.2020.00898 (PMC7422411; doi:10.3389/fgene.2020.00898)
Supplement: FILES 2 — Genomic sequences of the splice variants and the corresponding isoforms shown in Figure 6. [file Table_2.DOCX]

>SV1

ATGTTCAACCTCCCCAGGAGGCTGGAGGAGCTGCTGTGTCATCACGGTAGCATGCTGCCCAAGGGCGCAGACGAGGAGATACCACTCATCAAGCAAGATCTTGAGGAAATAATCTCAATCCTCCATGGTCACAGTGAGCCAAAACTGGAGGACCATAGCATGGTGGTCAGGTGCTGGATGAAGGAGGTGCGTGAGCTCTCTTACGACATCGAGGACAGTATTGACCAGTACGAGCATGCCGCCAGGTCTCAGAATAGACCTAATATTCACCACCGTAAGTTCAATCGGTGGCGTGGAAACAAGATCCCATGTATTCCCCAGAAACTGAAACAACGGTTGTGGATGGCCAACAAGATCAGAGAATTCAGCCTGCGCATCCAGGATGCGCTTCAACGCCATGCCATGTACAACAACCTCGGTGGTGTCGCTGGTACTGCTTCTACTACTAGAGGAGATGTATGCTCTGCCACACCTTGGCATCCCACAAAGACGCAGTTCAGAGAGCATGTCGACAACGTCCGTTCTGTCAGTATAGATGTCGATGGTATGGAAGCTGCCCTGAATGACTTGAACAAGCTCAAAAACTTGCTTGCTGGCATCCCGACTGCTTCTCTCGTGCAGTTCAGGGAGCATGCCGACAAAGTCCGTGGTATCCACACCGATATAGAAGCCATCCTGAACAAGCTCGAAAACATACCCCCTGGCATCACCACCACTACTACTACTACTACTAGAGAGGAGAAGCTCAAGGTGGTGTCCATCGTGGGAGTTGGAGGAGTTGGCAAGACTACGCTTGCCAACAAGCTGTACCGCAAGCTTCGGTGGCAGTTCGAGTGCCGGGCATTTGTGCGGACATCCCAGAAGACTGATATGAGGAGGCTTCTCATCAATATCCTCTTACAGATTCGGTCGCACCAATCACCTGACAATTGGAAGGTGCATAGCCTGATTTCCAGTATCAGGACATATCTGCAAGATAAGAGGTTCTTGATCGTAATTGATGATCTATGGGCTACATCCACATGGGATATTATTAAGTGCGCTTTGCCGGAGGGTAACAAGTCTAGCAGAATACTGACCACAACAGAAATTGAAGATCTAGCTTTGCAATCTTGTAGTTATGACTTGAAATTTATTTTCAAGATGATACCTTTTGGTGAGGATGACTCAAGAAAACTATTATTCAGTATAGTCTTTGGCTCTCATTCTAAGTGTCCTCCAGAAGTCAGTGAAACATTATATGATATTGTAAGGAAATGTGGTGGCTTGCCGCTAGCTATTGTCACTGTTGCAAGTCTTTTAGCAAGCCAGCTTGATAAACTGGAACAATGGGATTATATAAACAAATCCTTAGGTTACAGTTTGATGGCAAATCCTACTTTGGAAGGGATGAAACAACTACTGAACCTTTGTTACAACAATCTTCCTCAGCATTTGAAGGCATGCATGTTGTATCTTAGTATGTATCAAGGAGATCACATAATTTGGAAGGATGATTTAGTGAATCAATGGATAGCTGAAGGTTTTATCTGTGCAACTGAAGAGCATGACAAGGAAGAAATTTCAAGGGGCTATTTTGATGAGCTTGTTGGTAGAAAAATCATCCAGCCTGTCCATATCGATGACAGCGGTGAGGTTTTGTCCTGTGTAGTTCACCATATTGTACTCAATTTTGTTACATACAAGTCAATAGAAGAGAATTTTATTATTGCAATAGACCATTCACAGGCAACTATAAGATTTGCTGACAAGGTTCGACGATTATCTATTCACTTCAATAATGTAGAAGATGCACCTCCACCTACTAATATGAGATTGTTCCAAGTTCGGACAATTGCCTTCTTTGGGGTCTTGAAGTATATGCCTTTCATTATGGAGTTTCGACTTATTAAAGTTTTATTTCTACATTTTTTGGGTGATGAGGATAGCACCGGCATTGTTGATCTCACTAAAATTTCAGAACTTGTCCGACTGAGATATTTGAAGGTCACCTCTAATGCCACCGTAAAACTGCCAACCCGGTTGCAAGGTCTACCATATTTGGAGACACTGAAAATAGATGGAAAAATAAGTGAAGTTCCAACAGACATTTATTTGCCAGGTTTGCTGCATCTTACTCTTCCTGCTAAGACAAACCTGCCCAGTGGAATTGTCCACATGACATCGCTTCGTACAATTGAATATTTTGATCTCAGCTGTAACTCAGCGGAGAATCTATGGAGCCTTGGTGAGCTGAGCAATCTCCGGGATTTGCAGCTCACCTATTCTGAAATACATTCTGACAATCTGAAGGATAATATGAAATATCTTGGATCCATTCTGGGGAAACTCCGTAATCTCACATCTATAACTTTATCGCCTCCTGGCTCTTCCTGTCCAGATACTCTACATATTGACAGGGATACAAAGACGAGGATCAATGTTGATGGCTGGAGCAGTGTGTCCTCTCCACCAGCCCTTCTTCAGAGGTTTGAGTTGTTACCATGTGTTTGCATCTTTTCTAACCTCCCAAATTGGATTGGGCAGCTTGGAAACCTCTGCATTTTGAAGATTGGGATAAGGGAAGTAACAAGTAATAATATTGATGTTCTCGGAGTATTACCAGAGCTCACTGTTTTGTCACTTTATGTCCACACAAAGCCTGCAGAAAGGATTGTCTTTGACAATGCAGGGTTCTCAATCCTCAGATACTTCAAGTTTATATGCAGTGTAGCATGGATGAAATTTGAGGTTGGTGCAATGCCTAGTCTAAGGAAGCTCAAGTTAGGTTTTGATGTCCATAGAGCAGATCAGCATGATATTATTCCTGTTGGCATCGAACATCTGTCTGGACTTGAAGAGATCTCTGCCAAAATTAGGGTCGCTTCTACTGCTCATGATCATTGTAGAAGATTTGCAGAGTCAGCTTTGACTAACGCTATTAGGATGCATCCAGGACGTCCTAGCGTCAACATCCGATGTGTAGATTGGACCTTTGATGGTATGGATGTTAGCAATGCCGGGACACGGGAGGGAGAATGCAGGATTCTGAAAAAACAACAAAATATCGTGAAAGAAAGCTCTACTGAGAAGTCTGCAGTTCTAGAAAAGGATCGTGGGGATGGAGCAAATAAATCTGATGAAAGCAGGGAGAAGCTACCTATGGAAATGTGGCATGTGAGGAGTAATACGGAGGATGACGGCCTCAGCTGGAGTAAGTACGAGCAGAAGGAGATCCTCGGCGCCAAGTTCCCAAGAGCTTATTTCCGGTGCACACACTGGAACACGAAGAAGGGATGCATGGCGACCAAGGAGGTGCAGCGCGACGACGGTGACCCCCTCATGTTCGACATCGTATACCACGGTGAGCACACTTGCACTCAGACCGCGGAGTCCAATGTCGACGAACAGATCAGATTAACGCGAACGCGAGACAAGAAGGTAAAGAGAACGAAGAGGAAAAGGCAAGTGAGGGTGACCTCCGTGCCGGCGGATGACGGCTACAGCTGGAGGAAGTACGGGCAGAAGAACGTCCTCGGCTTCAGTTACCTAAGGTCAGTGGCTTACTATACGACGGTCGATCGATCGACTCACTGCAGCATCTCTGAAACGTACTGATTGCATGTGCATGATCGAGATTTAAACTGGCTAATATAAATCCATCCATCCATCGAACACTTGCGCACAGGGGTTACTACAGGTGTGCCACCAAGGGCTGCCAGGCGTCCAAGCAAGTGCAGCGCCACGACGACGGCTTGCTCTTCGACGTCACATACTTTGGTGAGCACACCTGCGCTGATCAGCCTCAGGCAGCGCACTCCAGCGACCAAGTACAGGTCACATTATGGCCGCCTGCCGTAAGCCCAGAGCAACCGCTCACACCGCAATCCGGGCTCGAGCAGAGCTCCACTGTCACTGTTACTGCATCAATACAGAGCACTACCCATAACTCTAGTATCATTGGGCCTAGAAGATCCAAACGAGAAGTCCACACCAACCCAAAGTATATGGGCTGTGATTGGGTTACTGGTTGA

>Isoform 1

MFNLPRRLEELLCHHGSMLPKGADEEIPLIKQDLEEIISILHGHSEPKLEDHSMVVRCWMKEVRELSYDIEDSIDQYEHAARSQNRPNIHHRKFNRWRGNKIPCIPQKLKQRLWMANKIREFSLRIQDALQRHAMYNNLGGVAGTASTTRGDVCSATPWHPTKTQFREHVDNVRSVSIDVDGMEAALNDLNKLKNLLAGIPTASLVQFREHADKVRGIHTDIEAILNKLENIPPGITTTTTTTTREEKLKVVSIVGVGGVGKTTLANKLYRKLRWQFECRAFVRTSQKTDMRRLLINILLQIRSHQSPDNWKVHSLISSIRTYLQDKRFLIVIDDLWATSTWDIIKCALPEGNKSSRILTTTEIEDLALQSCSYDLKFIFKMIPFGEDDSRKLLFSIVFGSHSKCPPEVSETLYDIVRKCGGLPLAIVTVASLLASQLDKLEQWDYINKSLGYSLMANPTLEGMKQLLNLCYNNLPQHLKACMLYLSMYQGDHIIWKDDLVNQWIAEGFICATEEHDKEEISRGYFDELVGRKIIQPVHIDDSGEVLSCVVHHIVLNFVTYKSIEENFIIAIDHSQATIRFADKVRRLSIHFNNVEDAPPPTNMRLFQVRTIAFFGVLKYMPFIMEFRLIKVLFLHFLGDEDSTGIVDLTKISELVRLRYLKVTSNATVKLPTRLQGLPYLETLKIDGKISEVPTDIYLPGLLHLTLPAKTNLPSGIVHMTSLRTIEYFDLSCNSAENLWSLGELSNLRDLQLTYSEIHSDNLKDNMKYLGSILGKLRNLTSITLSPPGSSCPDTLHIDRDTKTRINVDGWSSVSSPPALLQRFELLPCVCIFSNLPNWIGQLGNLCILKIGIREVTSNNIDVLGVLPELTVLSLYVHTKPAERIVFDNAGFSILRYFKFICSVAWMKFEVGAMPSLRKLKLGFDVHRADQHDIIPVGIEHLSGLEEISAKIRVASTAHDHCRRFAESALTNAIRMHPGRPSVNIRCVDWTFDGMDVSNAGTREGECRILKKQQNIVKESSTEKSAVLEKDRGDGANKSDESREKLPMEMWHVRSNTEDDGLSWSKYEQKEILGAKFPRAYFRCTHWNTKKGCMATKEVQRDDGDPLMFDIVYHGEHTCTQTAESNVDEQIRLTRTRDKKVKRTKRKRQVRVTSVPADDGYSWRKYGQKNVLGFSYLRSVAYYTTVDRSTHCSISETY

>SV2

ATGTTCAACCTCCCCAGGAGGCTGGAGGAGCTGCTGTGTCATCACGGTAGCATGCTGCCCAAGGGCGCAGACGAGGAGATACCACTCATCAAGCAAGATCTTGAGGAAATAATCTCAATCCTCCATGGTCACAGTGAGCCAAAACTGGAGGACCATAGCATGGTGGTCAGGTGCTGGATGAAGGAGGTGCGTGAGCTCTCTTACGACATCGAGGACAGTATTGACCAGTACGAGCATGCCGCCAGGTCTCAGAATAGACCTAATATTCACCACCGTAAGTTCAATCGGTGGCGTGGAAACAAGATCCCATGTATTCCCCAGAAACTGAAACAACGGTTGTGGATGGCCAACAAGATCAGAGAATTCAGCCTGCGCATCCAGGATGCGCTTCAACGCCATGCCATGTACAACAACCTCGGTGGTGTCGCTGGTACTGCTTCTACTACTAGAGGAGATGTATGCTCTGCCACACCTTGGCATCCCACAAAGACGCAGTTCAGAGAGCATGTCGACAACGTCCGTTCTGTCAGTATAGATGTCGATGGTATGGAAGCTGCCCTGAATGACTTGAACAAGCTCAAAAACTTGCTTGCTGGCATCCCGACTGCTTCTCTCGTGCAGTTCAGGGAGCATGCCGACAAAGTCCGTGGTATCCACACCGATATAGAAGCCATCCTGAACAAGCTCGAAAACATACCCCCTGGCATCACCACCACTACTACTACTACTACTAGAGAGGAGAAGCTCAAGGTGGTGTCCATCGTGGGAGTTGGAGGAGTTGGCAAGACTACGCTTGCCAACAAGCTGTACCGCAAGCTTCGGTGGCAGTTCGAGTGCCGGGCATTTGTGCGGACATCCCAGAAGACTGATATGAGGAGGCTTCTCATCAATATCCTCTTACAGATTCGGTCGCACCAATCACCTGACAATTGGAAGGTGCATAGCCTGATTTCCAGTATCAGGACATATCTGCAAGATAAGAGGTTCTTGATCGTAATTGATGATCTATGGGCTACATCCACATGGGATATTATTAAGTGCGCTTTGCCGGAGGGTAACAAGTCTAGCAGAATACTGACCACAACAGAAATTGAAGATCTAGCTTTGCAATCTTGTAGTTATGACTTGAAATTTATTTTCAAGATGATACCTTTTGGTGAGGATGACTCAAGAAAACTATTATTCAGTATAGTCTTTGGCTCTCATTCTAAGTGTCCTCCAGAAGTCAGTGAAACATTATATGATATTGTAAGGAAATGTGGTGGCTTGCCGCTAGCTATTGTCACTGTTGCAAGTCTTTTAGCAAGCCAGCTTGATAAACTGGAACAATGGGATTATATAAACAAATCCTTAGGTTACAGTTTGATGGCAAATCCTACTTTGGAAGGGATGAAACAACTACTGAACCTTTGTTACAACAATCTTCCTCAGCATTTGAAGGCATGCATGTTGTATCTTAGTATGTATCAAGGAGATCACATAATTTGGAAGGATGATTTAGTGAATCAATGGATAGCTGAAGGTTTTATCTGTGCAACTGAAGAGCATGACAAGGAAGAAATTTCAAGGGGCTATTTTGATGAGCTTGTTGGTAGAAAAATCATCCAGCCTGTCCATATCGATGACAGCGGTGAGGTTTTGTCCTGTGTAGTTCACCATATTGTACTCAATTTTGTTACATACAAGTCAATAGAAGAGAATTTTATTATTGCAATAGACCATTCACAGGCAACTATAAGATTTGCTGACAAGGTTCGACGATTATCTATTCACTTCAATAATGTAGAAGATGCACCTCCACCTACTAATATGAGATTGTTCCAAGTTCGGACAATTGCCTTCTTTGGGGTCTTGAAGTATATGCCTTTCATTATGGAGTTTCGACTTATTAAAGTTTTATTTCTACATTTTTTGGGTGATGAGGATAGCACCGGCATTGTTGATCTCACTAAAATTTCAGAACTTGTCCGACTGAGATATTTGAAGGTCACCTCTAATGCCACCGTAAAACTGCCAACCCGGTTGCAAGGTCTACCATATTTGGAGACACTGAAAATAGATGGAAAAATAAGTGAAGTTCCAACAGACATTTATTTGCCAGGTTTGCTGCATCTTACTCTTCCTGCTAAGACAAACCTGCCCAGTGGAATTGTCCACATGACATCGCTTCGTACAATTGAATATTTTGATCTCAGCTGTAACTCAGCGGAGAATCTATGGAGCCTTGGTGAGCTGAGCAATCTCCGGGATTTGCAGCTCACCTATTCTGAAATACATTCTGACAATCTGAAGGATAATATGAAATATCTTGGATCCATTCTGGGGAAACTCCGTAATCTCACATCTATAACTTTATCGCCTCCTGGCTCTTCCTGTCCAGATACTCTACATATTGACAGGGATACAAAGACGAGGATCAATGTTGATGGCTGGAGCAGTGTGTCCTCTCCACCAGCCCTTCTTCAGAGGTTTGAGTTGTTACCATGTGTTTGCATCTTTTCTAACCTCCCAAATTGGATTGGGCAGCTTGGAAACCTCTGCATTTTGAAGATTGGGATAAGGGAAGTAACAAGTAATAATATTGATGTTCTCGGAGTATTACCAGAGCTCACTGTTTTGTCACTTTATGTCCACACAAAGCCTGCAGAAAGGATTGTCTTTGACAATGCAGGGTTCTCAATCCTCAGATACTTCAAGTTTATATGCAGTGTAGCATGGATGAAATTTGAGGTTGGTGCAATGCCTAGTCTAAGGAAGCTCAAGTTAGGTTTTGATGTCCATAGAGCAGATCAGCATGATATTATTCCTGTTGGCATCGAACATCTGTCTGGACTTGAAGAGATCTCTGCCAAAATTAGGGTCGCTTCTACTGCTCATGATCATTGTAGAAGATTTGCAGAGTCAGCTTTGACTAACGCTATTAGGATGCATCCAGGACGTCCTAGCGTCAACATCCGATGTGTAGATTGGACCTTTGATGGTATGGATGTTAGCAATGCCGGGACACGGGAGGGAGAATGCAGGATTCTGAAAAAACAACAAAATATCGTGAAAGAAAGCTCTACTGAGAAGTCTGCAGTTCTAGAAAAGGATCGTGGGGATGGAGCAAATAAATCTGATGAAAGCAGGGAGAAGCTACCTATGGAAATGTGGCATGTGAGGAGTAATACGGAGGATGACGGCCTCAGCTGGAGTAAGTACGAGCAGAAGGAGATCCTCGGCGCCAAGTTCCCAAGGTCAGTGGGTGGGGTGGGTAACAATGACGGCACCTAAAACTTTTACTGGCACATACTCGCAGCTGTGTATACATGTACAATTTTTTCCGTCCTAAAATATTACTAACCTAAAATAGGATTTGCCAATCCTAAAACAACAAAGGTTGCTCTAAGGTGAATAAATCCCTTAAATCTTATCATTGGTTGCAACATTTTGGGATAGAGAAAACACTACTACTATCTCTGTTTTAAAATATTATTACCTAGTACATGATTGGATACTTTCTAGAGAATAAATTTTTTAATCATGTATTCCTTTTTTTCAATAAAAAAAAACCTAGTACACACAGCAGCTGGTCATACTAGGTAGCAATATTTTAATAAGGCGGAAGTAGTAGTATATGTGTGTACGCTTATAGTCTTAACACTTAATTGCAGAGCTTATTTCCGGTGCACACACTGGAACACGAAGAAGGGATGCATGGCGACCAAGGAGGTGCAGCGCGACGACGGTGACCCCCTCATGTTCGACATCGTATACCACGGTGAGCACACTTGCACTCAGACCGCGGAGTCCAATGTCGACGAACAGATCAGATTAACGCGAACGCGAGACAAGAAGGTAAAGAGAACGAAGAGGAAAAGGCAAGTGAGGGTGACCTCCGTGCCGGCGGATGACGGCTACAGCTGGAGGAAGTACGGGCAGAAGAACGTCCTCGGCTTCAGTTACCTAAGGTCAGTGGCTTACTATACGACGGTCGATCGATCGACTCACTGCAGCATCTCTGAAACGTACTGATTGCATGTGCATGATCGAGATTTAAACTGGCTAATATAAATCCATCCATCCATCGAACACTTGCGCACAGGGGTTACTACAGGTGTGCCACCAAGGGCTGCCAGGCGTCCAAGCAAGTGCAGCGCCACGACGACGGCTTGCTCTTCGACGTCACATACTTTGGTGAGCACACCTGCGCTGATCAGCCTCAGGCAGCGCACTCCAGCGACCAAGTACAGGTCACATTATGGCCGCCTGCCGTAAGCCCAGAGCAACCGCTCACACCGCAATCCGGGCTCGAGCAGAGCTCCACTGTCACTGTTACTGCATCAATACAGAGCACTACCCATAACTCTAGTATCATTGGGCCTAGAAGATCCAAACGAGAAGTCCACACCAACCCAAAGTATATGGGCTGTGATTGGGTTACTGGTTGA

>Isoform 2

MFNLPRRLEELLCHHGSMLPKGADEEIPLIKQDLEEIISILHGHSEPKLEDHSMVVRCWMKEVRELSYDIEDSIDQYEHAARSQNRPNIHHRKFNRWRGNKIPCIPQKLKQRLWMANKIREFSLRIQDALQRHAMYNNLGGVAGTASTTRGDVCSATPWHPTKTQFREHVDNVRSVSIDVDGMEAALNDLNKLKNLLAGIPTASLVQFREHADKVRGIHTDIEAILNKLENIPPGITTTTTTTTREEKLKVVSIVGVGGVGKTTLANKLYRKLRWQFECRAFVRTSQKTDMRRLLINILLQIRSHQSPDNWKVHSLISSIRTYLQDKRFLIVIDDLWATSTWDIIKCALPEGNKSSRILTTTEIEDLALQSCSYDLKFIFKMIPFGEDDSRKLLFSIVFGSHSKCPPEVSETLYDIVRKCGGLPLAIVTVASLLASQLDKLEQWDYINKSLGYSLMANPTLEGMKQLLNLCYNNLPQHLKACMLYLSMYQGDHIIWKDDLVNQWIAEGFICATEEHDKEEISRGYFDELVGRKIIQPVHIDDSGEVLSCVVHHIVLNFVTYKSIEENFIIAIDHSQATIRFADKVRRLSIHFNNVEDAPPPTNMRLFQVRTIAFFGVLKYMPFIMEFRLIKVLFLHFLGDEDSTGIVDLTKISELVRLRYLKVTSNATVKLPTRLQGLPYLETLKIDGKISEVPTDIYLPGLLHLTLPAKTNLPSGIVHMTSLRTIEYFDLSCNSAENLWSLGELSNLRDLQLTYSEIHSDNLKDNMKYLGSILGKLRNLTSITLSPPGSSCPDTLHIDRDTKTRINVDGWSSVSSPPALLQRFELLPCVCIFSNLPNWIGQLGNLCILKIGIREVTSNNIDVLGVLPELTVLSLYVHTKPAERIVFDNAGFSILRYFKFICSVAWMKFEVGAMPSLRKLKLGFDVHRADQHDIIPVGIEHLSGLEEISAKIRVASTAHDHCRRFAESALTNAIRMHPGRPSVNIRCVDWTFDGMDVSNAGTREGECRILKKQQNIVKESSTEKSAVLEKDRGDGANKSDESREKLPMEMWHVRSNTEDDGLSWSKYEQKEILGAKFPRSVGGVGNNDGT

>SV3

ATGTTCAACCTCCCCAGGAGGCTGGAGGAGCTGCTGTGTCATCACGGTAGCATGCTGCCCAAGGGCGCAGACGAGGAGATACCACTCATCAAGCAAGATCTTGAGGAAATAATCTCAATCCTCCATGGTCACAGTGAGCCAAAACTGGAGGACCATAGCATGGTGGTCAGGTGCTGGATGAAGGAGGTGCGTGAGCTCTCTTACGACATCGAGGACAGTATTGACCAGTACGAGCATGCCGCCAGGTCTCAGAATAGACCTAATATTCACCACCGTAAGTTCAATCGGTGGCGTGGAAACAAGATCCCATGTATTCCCCAGAAACTGAAACAACGGTTGTGGATGGCCAACAAGATCAGAGAATTCAGCCTGCGCATCCAGGATGCGCTTCAACGCCATGCCATGTACAACAACCTCGGTGGTGTCGCTGGTACTGCTTCTACTACTAGAGGAGATGTATGCTCTGCCACACCTTGGCATCCCACAAAGACGCAGTTCAGAGAGCATGTCGACAACGTCCGTTCTGTCAGTATAGATGTCGATGGTATGGAAGCTGCCCTGAATGACTTGAACAAGCTCAAAAACTTGCTTGCTGGCATCCCGACTGCTTCTCTCGTGCAGTTCAGGGAGCATGCCGACAAAGTCCGTGGTATCCACACCGATATAGAAGCCATCCTGAACAAGCTCGAAAACATACCCCCTGGCATCACCACCACTACTACTACTACTACTAGAGAGGAGAAGCTCAAGGTGGTGTCCATCGTGGGAGTTGGAGGAGTTGGCAAGACTACGCTTGCCAACAAGCTGTACCGCAAGCTTCGGTGGCAGTTCGAGTGCCGGGCATTTGTGCGGACATCCCAGAAGACTGATATGAGGAGGCTTCTCATCAATATCCTCTTACAGATTCGGTCGCACCAATCACCTGACAATTGGAAGGTGCATAGCCTGATTTCCAGTATCAGGACATATCTGCAAGATAAGAGGTTCTTGATCGTAATTGATGATCTATGGGCTACATCCACATGGGATATTATTAAGTGCGCTTTGCCGGAGGGTAACAAGTCTAGCAGAATACTGACCACAACAGAAATTGAAGATCTAGCTTTGCAATCTTGTAGTTATGACTTGAAATTTATTTTCAAGATGATACCTTTTGGTGAGGATGACTCAAGAAAACTATTATTCAGTATAGTCTTTGGCTCTCATTCTAAGTGTCCTCCAGAAGTCAGTGAAACATTATATGATATTGTAAGGAAATGTGGTGGCTTGCCGCTAGCTATTGTCACTGTTGCAAGTCTTTTAGCAAGCCAGCTTGATAAACTGGAACAATGGGATTATATAAACAAATCCTTAGGTTACAGTTTGATGGCAAATCCTACTTTGGAAGGGATGAAACAACTACTGAACCTTTGTTACAACAATCTTCCTCAGCATTTGAAGGCATGCATGTTGTATCTTAGTATGTATCAAGGAGATCACATAATTTGGAAGGATGATTTAGTGAATCAATGGATAGCTGAAGGTTTTATCTGTGCAACTGAAGAGCATGACAAGGAAGAAATTTCAAGGGGCTATTTTGATGAGCTTGTTGGTAGAAAAATCATCCAGCCTGTCCATATCGATGACAGCGGTGAGGTTTTGTCCTGTGTAGTTCACCATATTGTACTCAATTTTGTTACATACAAGTCAATAGAAGAGAATTTTATTATTGCAATAGACCATTCACAGGCAACTATAAGATTTGCTGACAAGGTTCGACGATTATCTATTCACTTCAATAATGTAGAAGATGCACCTCCACCTACTAATATGAGATTGTTCCAAGTTCGGACAATTGCCTTCTTTGGGGTCTTGAAGTATATGCCTTTCATTATGGAGTTTCGACTTATTAAAGTTTTATTTCTACATTTTTTGGGTGATGAGGATAGCACCGGCATTGTTGATCTCACTAAAATTTCAGAACTTGTCCGACTGAGATATTTGAAGGTCACCTCTAATGCCACCGTAAAACTGCCAACCCGGTTGCAAGGTCTACCATATTTGGAGACACTGAAAATAGATGGAAAAATAAGTGAAGTTCCAACAGACATTTATTTGCCAGGTTTGCTGCATCTTACTCTTCCTGCTAAGACAAACCTGCCCAGTGGAATTGTCCACATGACATCGCTTCGTACAATTGAATATTTTGATCTCAGCTGTAACTCAGCGGAGAATCTATGGAGCCTTGGTGAGCTGAGCAATCTCCGGGATTTGCAGCTCACCTATTCTGAAATACATTCTGACAATCTGAAGGATAATATGAAATATCTTGGATCCATTCTGGGGAAACTCCGTAATCTCACATCTATAACTTTATCGCCTCCTGGCTCTTCCTGTCCAGATACTCTACATATTGACAGGGATACAAAGACGAGGATCAATGTTGATGGCTGGAGCAGTGTGTCCTCTCCACCAGCCCTTCTTCAGAGGTTTGAGTTGTTACCATGTGTTTGCATCTTTTCTAACCTCCCAAATTGGATTGGGCAGCTTGGAAACCTCTGCATTTTGAAGATTGGGATAAGGGAAGTAACAAGTAATAATATTGATGTTCTCGGAGTATTACCAGAGCTCACTGTTTTGTCACTTTATGTCCACACAAAGCCTGCAGAAAGGATTGTCTTTGACAATGCAGGGTTCTCAATCCTCAGATACTTCAAGTTTATATGCAGTGTAGCATGGATGAAATTTGAGGTTGGTGCAATGCCTAGTCTAAGGAAGCTCAAGTTAGGTTTTGATGTCCATAGAGCAGATCAGCATGATATTATTCCTGTTGGCATCGAACATCTGTCTGGACTTGAAGAGATCTCTGCCAAAATTAGGGTCGCTTCTACTGCTCATGATCATTGTAGAAGATTTGCAGAGTCAGCTTTGACTAACGCTATTAGGATGCATCCAGGACGTCCTAGCGTCAACATCCGATGTGTAGATTGGACCTTTGATGGTATGGATGTTAGCAATGCCGGGACACGGGAGGGAGAATGCAGGATTCTGAAAAAACAACAAAATATCGTGAAAGAAAGCTCTACTGAGAAGTCTGCAGTTCTAGAAAAGGATCGTGGGGATGGAGCAAATAAATCTGATGAAAGCAGGGAGAAGCTACCTATGGAAATGTGGCATGTGAGGAGTAATACGGAGGATGACGGCCTCAGCTGGAGTAAGTACGAGCAGAAGGAGATCCTCGGCGCCAAGTTCCCAAGGTCAGTGGGTGGGGTGGGTAACAATGACGGCACCTAAAACTTTTACTGGCACATACTCGCAGCTGTGTATACATGTACAATTTTTTCCGTCCTAAAATATTACTAACCTAAAATAGGATTTGCCAATCCTAAAACAACAAAGGTTGCTCTAAGGTGAATAAATCCCTTAAATCTTATCATTGGTTGCAACATTTTGGGATAGAGAAAACACTACTACTATCTCTGTTTTAAAATATTATTACCTAGTACATGATTGGATACTTTCTAGAGAATAAATTTTTTAATCATGTATTCCTTTTTTTCAATAAAAAAAAACCTAGTACACACAGCAGCTGGTCATACTAGGTAGCAATATTTTAATAAGGCGGAAGTAGTAGTATATGTGTGTACGCTTATAGTCTTAACACTTAATTGCAGAGCTTATTTCCGGTGCACACACTGGAACACGAAGAAGGGATGCATGGCGACCAAGGAGGTGCAGCGCGACGACGGTGACCCCCTCATGTTCGACATCGTATACCACGGTGAGCACACTTGCACTCAGACCGCGGAGTCCAATGTCGACGAACAGATCAGATTAACGCGAACGCGAGACAAGAAGGTAAAGAGAACGAAGAGGAAAAGGCAAGTGAGGGTGACCTCCGTGCCGGCGGATGACGGCTACAGCTGGAGGAAGTACGGGCAGAAGAACGTCCTCGGCTTCAGTTACCTAAGGGGTTACTACAGGTGTGCCACCAAGGGCTGCCAGGCGTCCAAGCAAGTGCAGCGCCACGACGACGGCTTGCTCTTCGACGTCACATACTTTGGTGAGCACACCTGCGCTGATCAGCCTCAGGCAGCGCACTCCAGCGACCAAGTACAGGTCACATTATGGCCGCCTGCCGTAAGCCCAGAGCAACCGCTCACACCGCAATCCGGGCTCGAGCAGAGCTCCACTGTCACTGTTACTGCATCAATACAGAGCACTACCCATAACTCTAGTATCATTGGGCCTAGAAGATCCAAACGAGAAGTCCACACCAACCCAAAGTATATGGGCTGTGATTGGGTTACTGGTTGA

>Isoform 3

MFNLPRRLEELLCHHGSMLPKGADEEIPLIKQDLEEIISILHGHSEPKLEDHSMVVRCWMKEVRELSYDIEDSIDQYEHAARSQNRPNIHHRKFNRWRGNKIPCIPQKLKQRLWMANKIREFSLRIQDALQRHAMYNNLGGVAGTASTTRGDVCSATPWHPTKTQFREHVDNVRSVSIDVDGMEAALNDLNKLKNLLAGIPTASLVQFREHADKVRGIHTDIEAILNKLENIPPGITTTTTTTTREEKLKVVSIVGVGGVGKTTLANKLYRKLRWQFECRAFVRTSQKTDMRRLLINILLQIRSHQSPDNWKVHSLISSIRTYLQDKRFLIVIDDLWATSTWDIIKCALPEGNKSSRILTTTEIEDLALQSCSYDLKFIFKMIPFGEDDSRKLLFSIVFGSHSKCPPEVSETLYDIVRKCGGLPLAIVTVASLLASQLDKLEQWDYINKSLGYSLMANPTLEGMKQLLNLCYNNLPQHLKACMLYLSMYQGDHIIWKDDLVNQWIAEGFICATEEHDKEEISRGYFDELVGRKIIQPVHIDDSGEVLSCVVHHIVLNFVTYKSIEENFIIAIDHSQATIRFADKVRRLSIHFNNVEDAPPPTNMRLFQVRTIAFFGVLKYMPFIMEFRLIKVLFLHFLGDEDSTGIVDLTKISELVRLRYLKVTSNATVKLPTRLQGLPYLETLKIDGKISEVPTDIYLPGLLHLTLPAKTNLPSGIVHMTSLRTIEYFDLSCNSAENLWSLGELSNLRDLQLTYSEIHSDNLKDNMKYLGSILGKLRNLTSITLSPPGSSCPDTLHIDRDTKTRINVDGWSSVSSPPALLQRFELLPCVCIFSNLPNWIGQLGNLCILKIGIREVTSNNIDVLGVLPELTVLSLYVHTKPAERIVFDNAGFSILRYFKFICSVAWMKFEVGAMPSLRKLKLGFDVHRADQHDIIPVGIEHLSGLEEISAKIRVASTAHDHCRRFAESALTNAIRMHPGRPSVNIRCVDWTFDGMDVSNAGTREGECRILKKQQNIVKESSTEKSAVLEKDRGDGANKSDESREKLPMEMWHVRSNTEDDGLSWSKYEQKEILGAKFPRSVGGVGNNDGT

>SV4

ATGTTCAACCTCCCCAGGAGGCTGGAGGAGCTGCTGTGTCATCACGGTAGCATGCTGCCCAAGGGCGCAGACGAGGAGATACCACTCATCAAGCAAGATCTTGAGGAAATAATCTCAATCCTCCATGGTCACAGTGAGCCAAAACTGGAGGACCATAGCATGGTGGTCAGGTGCTGGATGAAGGAGGTGCGTGAGCTCTCTTACGACATCGAGGACAGTATTGACCAGTACGAGCATGCCGCCAGGTCTCAGAATAGACCTAATATTCACCACCGTAAGTTCAATCGGTGGCGTGGAAACAAGATCCCATGTATTCCCCAGAAACTGAAACAACGGTTGTGGATGGCCAACAAGATCAGAGAATTCAGCCTGCGCATCCAGGATGCGCTTCAACGCCATGCCATGTACAACAACCTCGGTGGTGTCGCTGGTACTGCTTCTACTACTAGAGGAGATGTATGCTCTGCCACACCTTGGCATCCCACAAAGACGCAGTTCAGAGAGCATGTCGACAACGTCCGTTCTGTCAGTATAGATGTCGATGGTATGGAAGCTGCCCTGAATGACTTGAACAAGCTCAAAAACTTGCTTGCTGGCATCCCGACTGCTTCTCTCGTGCAGTTCAGGGAGCATGCCGACAAAGTCCGTGGTATCCACACCGATATAGAAGCCATCCTGAACAAGCTCGAAAACATACCCCCTGGCATCACCACCACTACTACTACTACTACTAGAGAGGAGAAGCTCAAGGTGGTGTCCATCGTGGGAGTTGGAGGAGTTGGCAAGACTACGCTTGCCAACAAGCTGTACCGCAAGCTTCGGTGGCAGTTCGAGTGCCGGGCATTTGTGCGGACATCCCAGAAGACTGATATGAGGAGGCTTCTCATCAATATCCTCTTACAGATTCGGTCGCACCAATCACCTGACAATTGGAAGGTGCATAGCCTGATTTCCAGTATCAGGACATATCTGCAAGATAAGAGGTTCTTGATCGTAATTGATGATCTATGGGCTACATCCACATGGGATATTATTAAGTGCGCTTTGCCGGAGGGTAACAAGTCTAGCAGAATACTGACCACAACAGAAATTGAAGATCTAGCTTTGCAATCTTGTAGTTATGACTTGAAATTTATTTTCAAGATGATACCTTTTGGTGAGGATGACTCAAGAAAACTATTATTCAGTATAGTCTTTGGCTCTCATTCTAAGTGTCCTCCAGAAGTCAGTGAAACATTATATGATATTGTAAGGAAATGTGGTGGCTTGCCGCTAGCTATTGTCACTGTTGCAAGTCTTTTAGCAAGCCAGCTTGATAAACTGGAACAATGGGATTATATAAACAAATCCTTAGGTTACAGTTTGATGGCAAATCCTACTTTGGAAGGGATGAAACAACTACTGAACCTTTGTTACAACAATCTTCCTCAGCATTTGAAGGCATGCATGTTGTATCTTAGTATGTATCAAGGAGATCACATAATTTGGAAGGATGATTTAGTGAATCAATGGATAGCTGAAGGTTTTATCTGTGCAACTGAAGAGCATGACAAGGAAGAAATTTCAAGGGGCTATTTTGATGAGCTTGTTGGTAGAAAAATCATCCAGCCTGTCCATATCGATGACAGCGGTGAGGTTTTGTCCTGTGTAGTTCACCATATTGTACTCAATTTTGTTACATACAAGTCAATAGAAGAGAATTTTATTATTGCAATAGACCATTCACAGGCAACTATAAGATTTGCTGACAAGGTTCGACGATTATCTATTCACTTCAATAATGTAGAAGATGCACCTCCACCTACTAATATGAGATTGTTCCAAGTTCGGACAATTGCCTTCTTTGGGGTCTTGAAGTATATGCCTTTCATTATGGAGTTTCGACTTATTAAAGTTTTATTTCTACATTTTTTGGGTGATGAGGATAGCACCGGCATTGTTGATCTCACTAAAATTTCAGAACTTGTCCGACTGAGATATTTGAAGGTCACCTCTAATGCCACCGTAAAACTGCCAACCCGGTTGCAAGGTCTACCATATTTGGAGACACTGAAAATAGATGGAAAAATAAGTGAAGTTCCAACAGACATTTATTTGCCAGGTTTGCTGCATCTTACTCTTCCTGCTAAGACAAACCTGCCCAGTGGAATTGTCCACATGACATCGCTTCGTACAATTGAATATTTTGATCTCAGCTGTAACTCAGCGGAGAATCTATGGAGCCTTGGTGAGCTGAGCAATCTCCGGGATTTGCAGCTCACCTATTCTGAAATACATTCTGACAATCTGAAGGATAATATGAAATATCTTGGATCCATTCTGGGGAAACTCCGTAATCTCACATCTATAACTTTATCGCCTCCTGGCTCTTCCTGTCCAGATACTCTACATATTGACAGGGATACAAAGACGAGGATCAATGTTGATGGCTGGAGCAGTGTGTCCTCTCCACCAGCCCTTCTTCAGAGGTTTGAGTTGTTACCATGTGTTTGCATCTTTTCTAACCTCCCAAATTGGATTGGGCAGCTTGGAAACCTCTGCATTTTGAAGATTGGGATAAGGGAAGTAACAAGTAATAATATTGATGTTCTCGGAGTATTACCAGAGCTCACTGTTTTGTCACTTTATGTCCACACAAAGCCTGCAGAAAGGATTGTCTTTGACAATGCAGGGTTCTCAATCCTCAGATACTTCAAGTTTATATGCAGTGTAGCATGGATGAAATTTGAGGTTGGTGCAATGCCTAGTCTAAGGAAGCTCAAGTTAGGTTTTGATGTCCATAGAGCAGATCAGCATGATATTATTCCTGTTGGCATCGAACATCTGTCTGGACTTGAAGAGATCTCTGCCAAAATTAGGGTCGCTTCTACTGCTCATGATCATTGTAGAAGATTTGCAGAGTCAGCTTTGACTAACGCTATTAGGATGCATCCAGGACGTCCTAGCGTCAACATCCGATGTGTAGATTGGACCTTTGATGGTATGGATGTTAGCAATGCCGGGACACGGGAGGGAGAATGCAGGATTCTGAAAAAACAACAAAATATCGTGAAAGAAAGCTCTACTGAGAAGTCTGCAGTTCTAGAAAAGGATCGTGGGGATGGAGCAAATAAATCTGATGAAAGCAGGGAGAAGCTACCTATGGAAATGTGGCATGTGAGGAGTAATACGGAGGATGACGGCCTCAGCTGGAGTAAGTACGAGCAGAAGGAGATCCTCGGCGCCAAGTTCCCAAGAGCTTATTTCCGGTGCACACACTGGAACACGAAGAAGGGATGCATGGCGACCAAGGAGGTGCAGCGCGACGACGGTGACCCCCTCATGTTCGACATCGTATACCACGGTGAGCACACTTGCACTCAGACCGCGGAGTCCAATGTCGACGAACAGATCAGATTAACGCGAACGCGAGACAAGAAGGTAAAGAGAACGAAGAGGAAAAGGCAAGTGAGGGTGACCTCCGTGCCGGCGGATGACGGCTACAGCTGGAGGAAGTACGGGCAGAAGAACGTCCTCGGCTTCAGTTACCTAAGGGGTTACTACAGGTGTGCCACCAAGGGCTGCCAGGCGTCCAAGCAAGTGCAGCGCCACGACGACGGCTTGCTCTTCGACGTCACATACTTTGGTGAGCACACCTGCGCTGATCAGCCTCAGGCAGCGCACTCCAGCGACCAAGTACAGGTCACATTATGGCCGCCTGCCGTAAGCCCAGAGCAACCGCTCACACCGCAATCCGGGCTCGAGCAGAGCTCCACTGTCACTGTTACTGCATCAATACAGAGCACTACCCATAACTCTAGTATCATTGGGCCTAGAAGATCCAAACGAGAAGTCCACACCAACCCAAAGTATATGGGCTGTGATTGGGTTACTGGTTGA

>Isoform 4

MFNLPRRLEELLCHHGSMLPKGADEEIPLIKQDLEEIISILHGHSEPKLEDHSMVVRCWMKEVRELSYDIEDSIDQYEHAARSQNRPNIHHRKFNRWRGNKIPCIPQKLKQRLWMANKIREFSLRIQDALQRHAMYNNLGGVAGTASTTRGDVCSATPWHPTKTQFREHVDNVRSVSIDVDGMEAALNDLNKLKNLLAGIPTASLVQFREHADKVRGIHTDIEAILNKLENIPPGITTTTTTTTREEKLKVVSIVGVGGVGKTTLANKLYRKLRWQFECRAFVRTSQKTDMRRLLINILLQIRSHQSPDNWKVHSLISSIRTYLQDKRFLIVIDDLWATSTWDIIKCALPEGNKSSRILTTTEIEDLALQSCSYDLKFIFKMIPFGEDDSRKLLFSIVFGSHSKCPPEVSETLYDIVRKCGGLPLAIVTVASLLASQLDKLEQWDYINKSLGYSLMANPTLEGMKQLLNLCYNNLPQHLKACMLYLSMYQGDHIIWKDDLVNQWIAEGFICATEEHDKEEISRGYFDELVGRKIIQPVHIDDSGEVLSCVVHHIVLNFVTYKSIEENFIIAIDHSQATIRFADKVRRLSIHFNNVEDAPPPTNMRLFQVRTIAFFGVLKYMPFIMEFRLIKVLFLHFLGDEDSTGIVDLTKISELVRLRYLKVTSNATVKLPTRLQGLPYLETLKIDGKISEVPTDIYLPGLLHLTLPAKTNLPSGIVHMTSLRTIEYFDLSCNSAENLWSLGELSNLRDLQLTYSEIHSDNLKDNMKYLGSILGKLRNLTSITLSPPGSSCPDTLHIDRDTKTRINVDGWSSVSSPPALLQRFELLPCVCIFSNLPNWIGQLGNLCILKIGIREVTSNNIDVLGVLPELTVLSLYVHTKPAERIVFDNAGFSILRYFKFICSVAWMKFEVGAMPSLRKLKLGFDVHRADQHDIIPVGIEHLSGLEEISAKIRVASTAHDHCRRFAESALTNAIRMHPGRPSVNIRCVDWTFDGMDVSNAGTREGECRILKKQQNIVKESSTEKSAVLEKDRGDGANKSDESREKLPMEMWHVRSNTEDDGLSWSKYEQKEILGAKFPRAYFRCTHWNTKKGCMATKEVQRDDGDPLMFDIVYHGEHTCTQTAESNVDEQIRLTRTRDKKVKRTKRKRQVRVTSVPADDGYSWRKYGQKNVLGFSYLRGYYRCATKGCQASKQVQRHDDGLLFDVTYFGEHTCADQPQAAHSSDQVQVTLWPPAVSPEQPLTPQSGLEQSSTVTVTASIQSTTHNSSIIGPRRSKREVHTNPKYMGCDWVTG

**Domain positions**

SV1 6 128 Rx-CC like

SV1 262 474 NB-ARC

SV1 1059 1080 WRKY

SV2 6 128 Rx-CC like

SV2 262 474 NB-ARC

SV2 1059 1080 WRKY

SV3 6 128 Rx-CC like

SV3 262 474 NB-ARC

SV3 1059 1080 WRKY

SV4 6 128 Rx-CC like

SV4 262 474 NB-ARC

SV4 1059 1080 WRKY

SV4 1158 1213 WRKY

SV1 6 128 Rx-CC like

SV1 262 474 NB-ARC

SV1 1059 1080 WRKY

SV2 6 128 Rx-CC like

SV2 262 474 NB-ARC

SV2 1059 1080 WRKY

SV3 6 128 Rx-CC like

SV3 262 474 NB-ARC

SV3 1059 1080 WRKY

SV4 6 128 Rx-CC like

SV4 262 474 NB-ARC

SV4 1059 1080 WRKY

SV4 1158 1213 WRKY
