# Supplementary material for: Wheat Disease Resistance Genes and Their Diversification Through Integrated Domain Fusions
Source: Front Genet. 2020 Aug 5;11:898. doi: 10.3389/fgene.2020.00898 (PMC7422411; doi:10.3389/fgene.2020.00898)
Supplement: Supplementary file 3 [file Presentation_1.PPTX]

## Slide 1
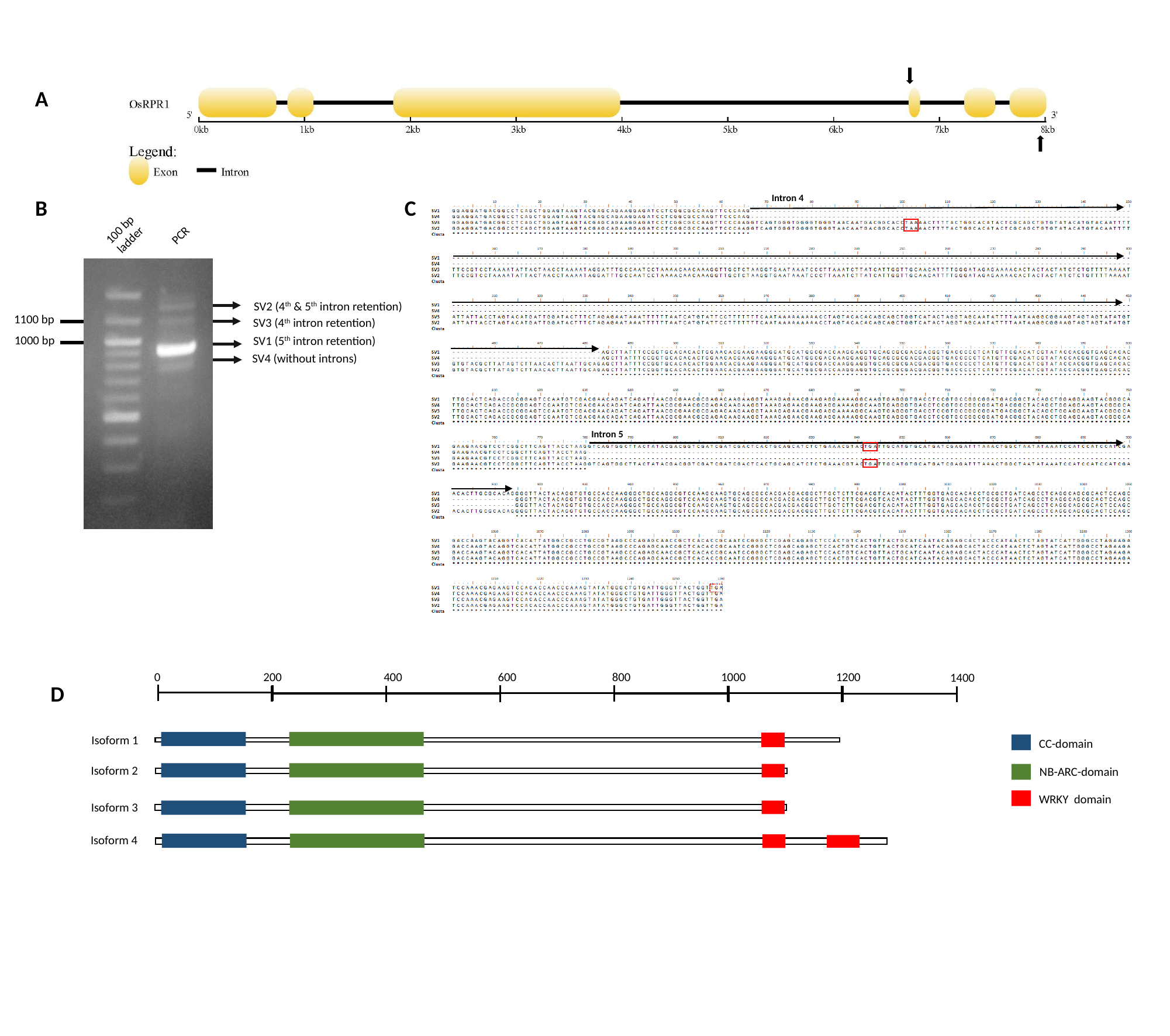

A
Intron 4
B
C
100 bp ladder
PCR
SV2 (4th & 5th intron retention)
1100 bp
SV3 (4th intron retention)
 1000 bp
SV1 (5th intron retention)
SV4 (without introns)
Intron 5
0
400
800
1200
200
600
1000
1400
D
Isoform 1
CC-domain
Isoform 2
NB-ARC-domain
WRKY domain
Isoform 3
Isoform 4
